# Three New Triterpene Esters from Pumpkin (*Cucurbita maxima*) Seeds

**DOI:** 10.3390/molecules19044802

**Published:** 2014-04-16

**Authors:** Takashi Kikuchi, Shinsuke Ueda, Jokaku Kanazawa, Hiroki Naoe, Takeshi Yamada, Reiko Tanaka

**Affiliations:** Osaka University of Pharmaceutical Sciences, 4-20-1 Nasahara, Takatsuki, Osaka, 569-1094, Japan

**Keywords:** *Cucurbita maxima*, multiflorane-type triterpene, melanogenesis inhibitory activity, cytotoxic activity

## Abstract

Three new multiflorane-type triterpene esters, *i.e.* 7α-hydroxymultiflor-8-ene-3α,29-diol 3-acetate-29-benzoate (**1**), 7α-methoxymultiflor-8-ene-3α,29-diol 3,29-dibenzoate (**2**), and 7β-methoxymultiflor-8-ene-3α,29-diol 3,29-dibenzoate (**3**), were isolated from seeds of *Cucurbita maxima*, along with the known compound, multiflora-7,9(11)-diene-3α,29-diol 3,29-dibenzoate (**4**). Compound **1** exhibited melanogenesis inhibitory activities comparable with those of arbutin. In cytotoxicity assays, compounds **1** and **3** exhibited weak cytotoxicity, with IC_50_ values of 34.5–93.7 μM against HL-60 and P388 cells.

## 1. Introduction

Pumpkins, including *Cucurbita moschata*, *C. pepo*, and *C. maxima*, are gourd squashes of the genus *Cucurbita* and the family Cucurbitaceae. *Cucurbita moschata* seeds have been used as an anthelmintic [1], and *Cucurbita pepo* seeds, as an anthelmintic and a diuretic [2]. The isolation of 3-*p*-aminobenzoyl multiflorane-type triterpenes, namely 3-*O*-*p*-aminobenzoyl-29-*O*-benzoylmultifrora-8-ene-3α,7β,29-triol and 3-*O*-*p*-aminobenzoyl-29-*O*-benzoylmultifrora-7,9(11)-diene-3α,29-diol, and 7-*epi* zucchini factor A, and debenzoyl zucchini factor B from *C. pepo* seeds has been reported [3,4].

*Cucurbita maxima* (English name: squash, pumpkin, Japanese name: Kabocha) is indigenous to the plateaus of central and south America, but is nowadays cultivated throughout the world. Its fruits, flowers, and seeds have been eaten as vegetables containing vitamins A, C, and E. Several triterpenes, such as cucurbita-5,24-dienol [5] and α- and β-amyrin [6], are present in the seeds of *Cucurbita maxima*. Additionally, it was demonstrated that the seeds and flowers of *Cucurbita maxima* contain sterols [6,7,8]. Recently we have reported the isolation of six multiflorane-type triterpenes including three new triterpenes: 7α-methoxymultiflor-8-ene-3α,29-diol 3-acetate-29-benzoate, 7-oxomultiflor-8-ene-3α,29-diol 3-acetate-29-benzoate, and multiflora-7,9(11)-diene-3α,29-diol 3-*p*-hydroxybenzoate-29-benzoate, from seeds of *C. maxima* produced in Japan, and the melanogenesis inhibitory and cytotoxic activities of these compounds [9]. In a continuing study to explore new compounds possessing potent biological activities from *C. maxima* seeds, we have isolated four multiflorane-type triterpenes from seeds of *C. maxima* produced in India, and determined the structures of three new compounds: 7α-hydroxymultiflor-8-ene-3α,29-diol 3-acetate-29-benzoate (**1**) 7α-methoxymultiflor-8-ene-3α,29-diol 3,29-dibenzoate (**2**), and 7β-methoxymultiflor-8-ene-3α,29-diol 3,29-dibenzoate (**3**). In addition, **1**–**3**, were evaluated for inhibitory effects on α-MSH-induced melanogenesis in B16 melanomas, and cytotoxic activities against the HL-60 and P388 leukemia cell lines. 

## 2. Results and Discussion

**Figure 1:**
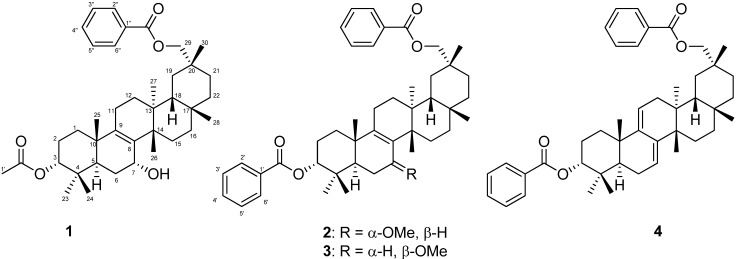
Chemical structures of isolated compounds **1**−**4**.

Four multiflorane-type triterpenes, including three new compounds, *i.e.* 7α-hydroxymultiflor-8-ene-3α,29-diol 3-acetate-29-benzoate (**1**), 7α-methoxymultiflor-8-ene-3α,29-diol 3,29-dibenzoate (**2**), and 7β-methoxymultiflor-8-ene-3α,29-diol 3,29-dibenzoate (**3**), were isolated from the MeOH extract of *C. maxima* seeds (Figure 1). The known compound, *i.e.* multiflora-7,9(11)-diene-3α,29-diol 3,29-dibenzoate (**4**), was identified by comparing its MS and ^1^H-NMR data with published values [10].

**Figure 2:**
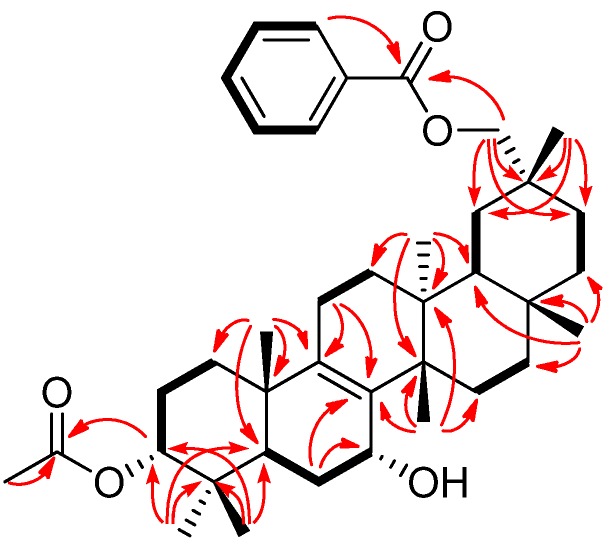
Key HMBC and ^1^H-^1^H COSY correlations of compound **1**.

**Figure 3:**
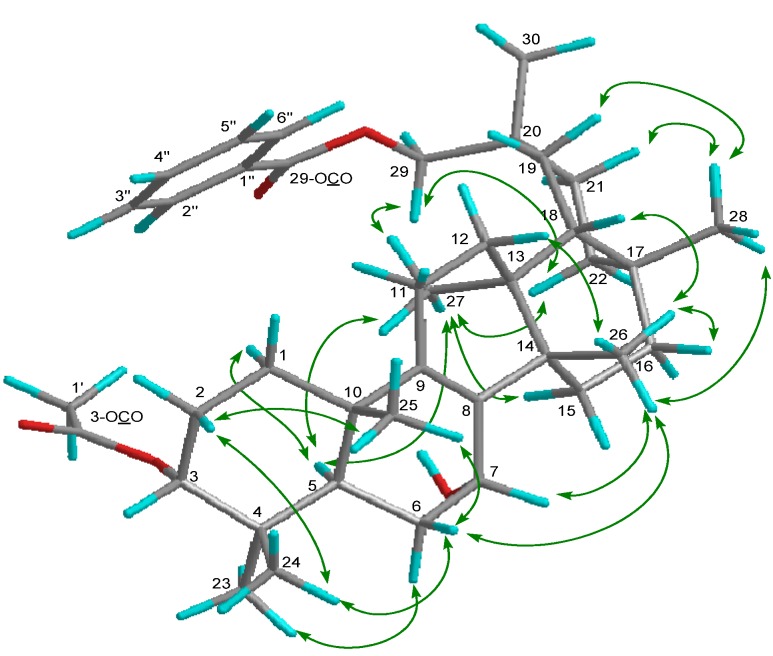
Selected NOE correlations of compound **1**.

Compound **1 **exhibited a [M–H_2_O]^+^ ion in the HREIMS data at *m/z* 586.4019 compatible with the molecular formula C_39_H_54_O_4 _(calcd. 586.4023), therefore it was suggested that the molecular formula of **1** is C_39_H_56_O_5_. The IR spectrum showed the presence of a hydroxy group (ν_max_ 3437 cm^−1^) and ester carbonyl groups (ν_max_ 1718, 1272, 1244 cm^−1^). The ^1^H and ^13^C-NMR spectra (Table 1) displayed signals for seven tertiary methyl groups [δ_H_ 0.89, 0.91, 0.92, 1.03, 1.06, 1.10, 1.12 (each s)], an oxymethylene [δ_H_ 4.14 (2H, brs); δ_C_ 72.5 (t)], two oxymethines [δ_H_ 4.16 (brs), 4.69 (t); δ_C_ 64.3 (d), 77.4 (d)], a tetrasubstituted olefin [δ_C_ 136.4 (s), 140.1 (s)], an acetoxy group [δ_H_ 2.07 (s); δ_C_ 21.4 (q), 170.9 (s)], and a benzoyl group [δ_H_ 7.47 (tt), 7.58 (tt), 8.07 (dd); δ_C_ 128.4 (d), 129.4 (d), 130.6 (s), 132.9 (d), 166.7 (s)]. The ^1^H- and ^13^C-NMR spectra are similar to those of 3-*O*-*p*-aminobenzoyl-29-*O*-benzoylmultiflor-8-ene-3α,7α,29-triol [3] except for the absence of a *p*-aminobenzoyl group at C-3 and existence of an acetyl group in **1**. In the HMBC experiment, the following correlations were observed: Me-23 [δ_H_ 0.89 (s)] to C-3 [δ_c_ 77.4 (d)], C-4, C-5, and C-24; Me-24 [δ_H_ 0.91 (s)] to C-3, C-4, C-5, and Me-23; Me-25 [δ_H_ 0.92 (s)] to C-1, C-5, C-9 [δ_C_ 140.1 (s)], and C-10; Me-26 [δ_H_ 1.03 (s)] to C-8 [δ_C_ 136.4 (s)], C-13, C-14, and C-15; Me-27 [δ_H_ 1.06 (s)] to C-12, C-13, C-14, and C-18; Me-28 [δ_H_ 1.12 (s)] to C-16, C-17, C-18, and C-22; H_2_-29 [δ_H_ 4.14 (2H, brs)] to C-19, C-20, C-21, C-30, and 29-OCO [δ_C_ 166.7 (s)]; Me-30 [δ_H_ 1.10 (s)] to C-19, C-20, C-21, and C-29; H-3 [δ_H_ 4.69 (t)] to 3-OCO [δ_C_ 170.9 (s)]; H-5 and H-6α to C-7 [δ_C_ 64.3 (d)]; H-6α and H_2_-11 to C-8 [δ_C_ 136.4 (s)]; H-11 and H-12δ to C-9 [δ_C_ 140.1 (s)]. In the ^1^H-^1^H COSY experiment, H-7 [δ_H_ 4.16 (brs)] correlated with H_2_-6 [δ_H_ 1.60, 1.74] (Figure 2). The following significant NOE interactions were observed in **1**: H-5/H-1α, Me-27; Me-23/H-6α; Me-27/H-15α, H-22α, and H_2_-29; H-2β /Me-24, and Me-25; Me-25/H-6β; Me-26/H-6β, H-7β, H-12β, H-16β, and Me-28; Me-28/H-19β, H-21β (Figure 3). In addition, the NOE correlations between H-7 and Me-26 suggested that the hydroxy group at C-7 is in the α (axial)-orientation (Figure 3). The configuration of the acetoxy group at C-3 was established as the α (axial)-orientation due to the coupling constants of H-3 [δ_H_ 4.69 (t, *J*_3β.2α;3β,2β_ = 3.0 Hz)] and NOEs between H-3 and Me-24. Therefore, the structure of **1** was determined to be as shown in Figure 1.

**Table 1:** ^1^H (600 MHz) and ^13^C (150 MHz) NMR spectroscopic data of compounds **1**–**3** (CDCl_3_) ^a^.

	1					2					3				
Position	δ_C_, type			δ_H_ (*J* in Hz)		δ_C_, type			δ_H_ (*J* in Hz)		δ_C_, type			δ_H_ (*J* in Hz)	
1	29.6	t	α	1.33	m	29.8	t	α	1.48	m	30.6	t	α	1.40	m
			β	1.47	m			β	1.53	m			β	1.52	m
2	23.3	t	α	1.66	m	23.6	t	α	1.80	m	23.3	t	α	1.80	m
			β	1.88	m			β	1.98	m			β	1.97	m
3	77.4	d		4.69	t (3.0)	78.1	d		4.95	t (2.9)	78.2	d		4.91	t (3.0)
4	36.3	s				36.7	s				37.0	s			
5	39.6	d		1.91	m	39.9	d		2.19	dd (12.6, 1.2)	44.1	d		1.70	m
6	28.8	t	α	1.74	m	22.5	t	α	1.95	m	25.3	t	α	2.21	m
			β	1.60	m			β	1.34	m			β	1.51	m
7	64.3	d		4.16	brs	73.8	d		3.54	brs	78.8	d		3.98	brt (7.6)
8	136.4	s				135.3	s				136.7	s			
9	140.1	s				139.7	s				140.3	s			
10	38.6	s				38.6	s				38.2	s			
11	20.9	t		1.93	2H, m	20.9	t		1.97	2H, m	20.8	t	α	2.03	m
													β	1.91	m
12	31.19	t	α	1.34	m	31.3	t	α	1.35	m	30.7	t	α	1.51	m
			β	1.61	m			β	1.61	m			β	1.40	m
13	36.8	s				37.0	s				38.2	s			
14	41.9	s				41.8	s				40.9	s			
15	26.1	t	α	2.05	m	25.4	t	α	2.19	m	26.3	t	α	1.78	m
			β	1.50	m			β	1.26	m			β	1.83	m
16	36.9	t	α	1.54	m	36.9	t	α	1.56	m	36.5	t	α	1.61	m
			β	1.67	m			β	1.61	m			β	1.53	m
17	31.2	s				31.1	s				31.2	s			
18	44.4	d		1.61	m	44.0	d		1.60	m	42.8	d		1.66	m
19	28.4	t	α	1.90	m	28.8	t	α	1.86	m	29.8	t	α	1.40	m
			β	1.56	m			β	1.49	m			β	1.50	m
20	31.7	s				31.9	s				32.2	s			
21	30.1	t	α	1.47	m	29.9	t	α	1.48	m	29.1	t		1.52	2H, m
			β	1.59	m			β	1.53	m					
22	35.0	t	α	1.87	m	35.6	t	α	1.84	d (4.4)	37.1	t	α	1.68	m
			β	0.97	m			β	0.96	m			β	1.01	m
23	27.4	q		0.89	s	27.5	q		0.97	s	27.6	q		0.96	s
24	22.1	q		0.91	s	22.4	q		1.00	s	21.7	q		1.02	s
25	18.1	q		0.92	s	18.2	q		0.98	s	20.2	q		1.10	s
26	25.1	q		1.03	s	26.0	q		1.05	s	27.8	q		1.29	s
27	19.0	q		1.06	s	19.0	q		1.082	s	18.0	q		0.95	s
28	31.17	q		1.12	s	31.3	q		1.13	s	30.7	q		1.17	s
29	72.5	t		4.14	2H, brs	72.9	t	A	4.08	d (10.8)	74.0	t	A	4.05	d (10.6)
								B	4.16	d (10.8)			B	4.11	d (10.6)
30	30.6	q		1.10	s	29.8	q		1.084	s	28.1	q		1.12	s
3-OCO	170.9	s				166.3	s				165.9	s			
1'	21.4	q		2.07	s	130.8^b^	s				130.6	s			
2', 6'						129.6^c^	d		8.05 ^b^	dd (7.4, 1.4)	129.4	d		7.99	dd (7.4, 1.4)
3', 5'						128.4^d^	d		7.45 ^c^	tt (7.4, 1.4)	128.5	d		7.45	tt (7.4, 1.4)
4'						132.7^e^	d		7.55 ^d^	tt (7.4, 1.4)	132.8	d		7.56	tt (7.4, 1.4)
29-OCO	166.7	s				166.6	s				166.8	s			
1''	130.6	s				130.7^b^	s				130.9	s			
2'', 6''	129.4	d		8.07	dd (7.4, 1.2)	129.4^c^	d		8.04 ^b^	dd (7.4, 1.4)	129.5	d		8.04	dd (7.3, 1.7)
3'', 5''	128.4	d		7.47	tt (7.4, 1.2)	128.3^d^	d		7.43 ^c^	tt (7.4, 1.4)	128.4	d		7.43	tt (7.3, 1.7)
4''	132.9	d		7.58	tt (7.4, 1.2)	132.6^e^	d		7.54 ^d^	tt (7.4, 1.4)	132.7	d		7.55	tt (7.3, 1.7)
7-OMe						54.9	q		3.24	s	55.0	q		3.35	s

^a^ Assignments were based on ^1^H-^1^H COSY, HMQC, HMBC and NOESY supectroscopic data. ^b−e^ Interchengeable.

**Figure 4:**
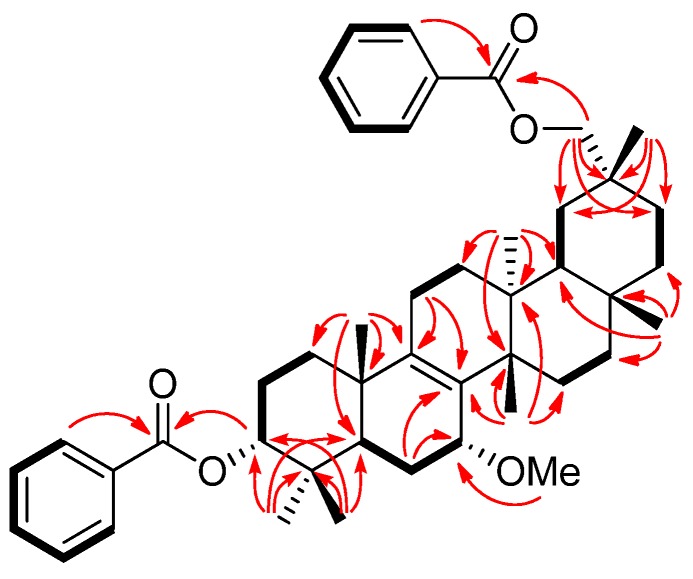
Key HMBC and ^1^H-^1^H COSY correlations of compound **2**.

**Figure 5:**
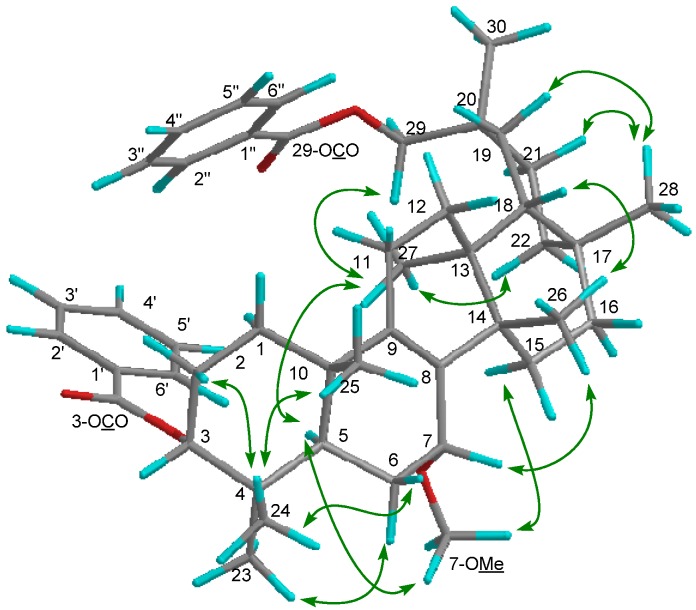
Selected NOE correlations of compound **2**.

Compound **2** exhibited a [M]^+^ ion in the HREIMS data at *m/z* 680.4447 compatible with the molecular formula C_45_H_60_O_5_ (calcd. 680.4441). The IR spectrum showed absorption indicating ester carbonyl groups (ν_max_ 1717, 1274 cm^−1^). The ^1^H- and ^13^C-NMR spectra (Table 1) displayed signals for seven tertiary methyl groups [δ_H_ 0.97, 0.98, 1.00, 1.05, 1.082, 1.084, 1.13 (each s)], an oxymethylene [δ_H_ 4.08, 4.16 (each d); δ_C_ 72.9 (t)], two oxymethines [δ_H_ 3.54 (brs), 4.95 (t); δ_C_ 73.8 (d), 78.1 (d)], a tetrasubstituted olefin [δ_C_ 135.3 (s), 139.7 (s)], and two benzoyl groups [δ_H_ 7.43 (tt), 7.45 (tt), 7.54 (tt), 7.55 (tt), 8.04 (dd), 8.05 (dd); δ_C_ 128.3 (d), 128.4 (d), 129.4 (d), 129.6 (d), 130.7 (s), 130.8 (s), 132.6 (d), 132.7 (d), 166.3 (s), 166.6 (s)]. The above data suggested that the structure of **2** is similar to that of 7α-methoxymultiflor-8-ene-3α,29-diol 3-acetate-29-benzoate [9], except for the lack of the 3-*O*-acetyl group and the existence of a 3-*O*-benzoyl group. In the HMBC experiment, the following correlations were observed: H_2_-29 [δ_H_ 4.08, 4.16 (each d)] to 29-OCO [δ_C_ 166.6 (s)]; H-3 [δ_H_ 4.95 (t)] to 3-OCO [δ_C_ 166.3 (s)]; H-5 and H-6α to C-7 [δ_C_ 73.8 (d)]; H-6α, H-7β, and Me-26 to C-8 [δ_C_ 135.3 (s)]; H-7β, H_2_-11 and Me-25 to C-9 [δ_C_ 139.7 (s)]. In the ^1^H-^1^H COSY experiment, H-7 [δ_H_ 3.54 (brs)] correlated with H_2_-6 [δ_H_ 1.34, 1.95] (Figure 4). Additionally, the NOEs were observed H-7/H-15β, Me-26; 7-OMe/H-5 and H-15α; suggested that the methoxy group at C-7 was in the α-orientation (Figure 5). The configuration of the acetoxy group at C-3 was established as the α-orientation due to the significant NOEs between H-3 and Me-24, and the coupling constants of H-3 [δ_H_ 4.95 (t, *J* = 2.9 Hz)]. Therefore, **2** was established as shown in Figure 1.

**Figure 6:**
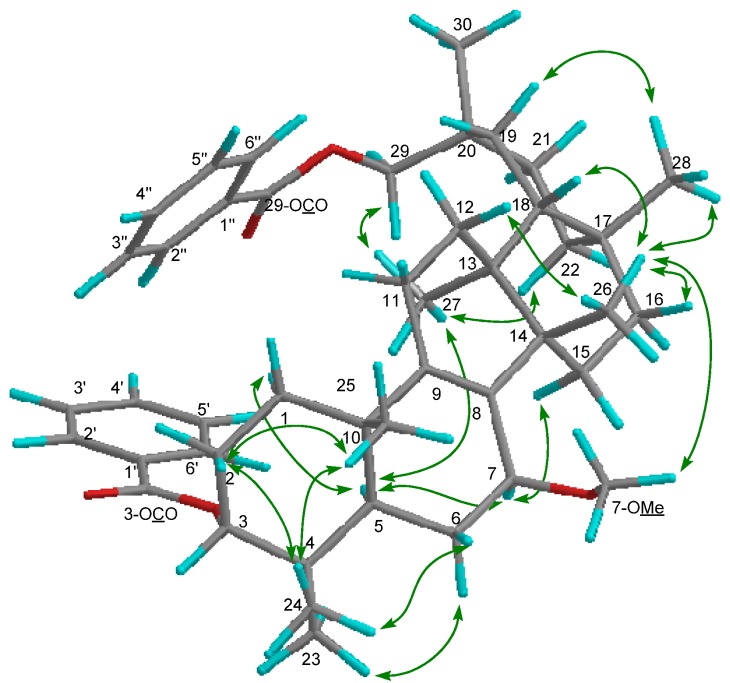
Selected NOE correlations of compound **3**.

Compound **3** exhibited a [M]^+^ ion in the HREIMS data at *m/z* 680.4446 compatible with the molecular formula C_45_H_60_O_5_ (calcd. 680.4440). The IR spectrum showed absorption indicating ester carbonyl groups (ν_max_ 1717, 1272 cm^−1^). The ^1^H- and ^13^C-NMR spectra (Table 1) displayed signals for seven tertiary methyl groups [δ_H_ 0.95, 0.96, 1.02, 1.10, 1.12, 1.17, 1.29 (each s)], an oxymethylene [δ_H_ 4.05, 4.11 (each d); δ_C_ 74.0 (t)], two oxymethines [δ_H_ 3.98 (brt), 4.91 (t); δ_C_ 78.2 (d), 78.8 (d)], a tetrasubstituted olefin [δ_C_ 136.7 (s), 140.3 (s)], and two benzoyl group [δ_H_ 7.43 (tt), 7.45 (tt), 7.55 (tt), 7.56 (tt), 7.99 (dd), 8.04 (dd); δ_C_ 128.4 (d), 128.5 (d), 129.4 (d), 129.5 (d), 130.6 (s), 130.9 (s), 132.7 (d), 132.8 (d), 165.9 (s), 166.8 (s)]. The ^1^H- and ^13^C-NMR spectra of **3** were very similar to those of **2**, except for the H-7 signal [δ_H_ 3.98 (brt, *J* = 7.6 Hz): δ_C_ 78.8 (d) in **3**; δ_H_ 3.54 (brs): δ_C_ 73.8 (d) in **2**]. The coupling constants of H-7, and the NOE correlations of H-7/H-5α, and H-15α; 7-OMe/Me-26 suggested that the methoxy group at C-7 is in the β (equatorial)-orientation (Figure 6). The configuration of the benzoyl group at C-3 was established as the α-orientation due to the coupling constants of H-3 [δ_H_ 4.91 (t, *J* = 3.0 Hz)]. The above data established that **3** was a 7β-methoxy epimer of **2** (Figure 1).

Multiflorane-type triterpenes are unusual, and most of them have been isolated from cucurbitaceae plants, such as *Cucumis melo* [11], *Cucurbita pepo* [3,4], *Momordica cochinchinensis* [12], and *Trichosanthes kirilowii* [13]. Only a few of their biological activities, such as anti-tumor promoting activities [14], anti-oxidant effects [15], cytotoxic activities [9,16], and melanogenesis inhibitory activities [9,16], have been reported. In this study, we evaluated them for melanogenesis inhibitory effects and cytotoxic activities against cancer cell lines. Melanogenesis plays an important role to protect the skin from UV irradiation. However, overproduction of melanin causes esthetic and dermatological problems [17], thus, several hypopigmenting products have been developed [17]. In this study, three new multiflorane triterpenes **1**–**3** from *C. maxima* were evaluated for inhibitory activities against α-MSH-induced melanogenesis in B16 melanomas (Table 2). To determine the safe concentration, cytotoxicities of compounds against B16 4A5 cells were examined by an MTT assay. Compound **2** did not exhibit cytotoxicity at 10–100 μM. Compounds **1** and **3** showed no toxicities at 10 μM, although they decreased cell viabilities at higher concentrations (**1**: 88.0% at 30 μM, 58.4% at 100 μM; **3**: 86.3% at 30 μM, 67.2% at 100 μM). In the melanogenesis inhibitory assay, compound **1** reduced melanin content (88.5%) at a non-toxic concentration, 10 μM. The melanogenesis inhibitory activity of compound **1** was comparable with that of the positive control, arbutin (melanin content 88.9% at 10 μM), which has been recognized as a useful depigmentation compound for skin whitening in the cosmetic industry [18]. These results suggested that compound **2** may be valuable as a potential skin-whitening agent. Compounds **2** (10–100 μM) and **3** (10 μM) did not show any melanogenesis inhibitory activities.

**Table 2:** Melanogenesis inhibitory activity and cytotoxicity in B16 mouse melanoma cells of multiflorane-type triterpenes isolated from *Cucurbita maxima* seeds ^a^.

Compound		Conc. (μM)				IC_50_ (μM)
		10	30	100	300	
**1**	melanin content	88.5 ± 2.7 **	65.8 ± 1.7 **	35.7 ± 0.8 **		46.5
	cell viability	105.4 ± 4.5	88.0 ± 1.0	58.4 ± 7.7 **		>100
**2**	melanin content	96.3 ± 2.2	105.5 ± 4.5	108.3 ± 1.0		>100
	cell viability	103.4 ± 3.6	105.8 ± 5.0	104.9 ± 7.7		>100
**3**	melanin content	96.9 ± 9.2	79.4 ± 4.5 **	60.1 ± 1.8 **		>100
	cell viability	94.6 ± 0.4	86.3 ± 2.4 *	67.2 ± 4.8**		>100
**4** ^b^	melanin content	98.4 ± 3.2	102.2 ± 11.7	95.4 ± 8.4		>100
	cell viability	110.8 ± 4.3	103.0 ± 8.2	101.1 ± 5.9		>100
Arbutin ^c^	melanin content	88.9 ± 2.3 **	72.3 ± 3.1 **	55.3 ± 1.0 **	33.8 ± 2.8 **	124.6
	cell viability	100.0 ± 2.7	94.4 ± 1.2	89.9 ± 0.3 **	81.9 ± 3.2 **	>300

^a^ Melanin content (%) and cell viability (%) were determined based on the absorbance at 450 nm, and 540 nm, respectively, by comparison with values for DMSO (100%). Each value represents the mean ± standard deviation (S.D.) of three determinations. Asterisks denote significant differences from control group, * *p* < 0.05, ** *p* < 0.01. The concentration of DMSO in the sample solution was 2 μL/mL. ^b^ Melanogenesis inhibitory and cytotoxicity data from [9]. ^c^ Reference compound.

Three triterpenes and a reference compound, 5-fluorouracil (5-FU), were also evaluated for cytotoxic activities against human leukemia (HL-60) and murine leukemia (P388) cell lines by means of the MTT assay. Compounds **1** and **3** exhibited weak cytotoxicities against HL-60 (IC_50_ **1**:89.2 μM; **3**:64.6 μM) and P388 (IC_50_ **1**:93.7 μM; **3**:34.5 μM). Compound **2** did not show activities against either cell line (IC_50_ each > 100 μM). In our previous study, several mutiflorane-type triterpenes were evaluated for their cytotoxic activities, and they showed no or weak activities, except 7-oxomultiflor-8-ene-3α,29-diol 3-acetate-29-benzoate, having a conjugated enone [9]. Results of this and previous studies suggest that a conjugated enone moiety strengthens the cytotoxic activities of multiflorane-type triterpenes.

## 3. Experimental

### 3.1. General Experimental Procedures

Chemicals and reagents were purchased as follows: fetal bovine serum (FBS) from Invitrogen (Carlsbad, CA, USA), 3-(4,5-dimethyl-2-thiazolyl)-2,5-diphenyl-2*H*-tetrazolium bromide (MTT) from Sigma-Aldrich Japan Co*.* (Tokyo, Japan), Roswell Park Memorial Institute (RPMI) 1640 medium, Dulbecco’s modified Eagle’s medium (D-MEM), and antibiotics from Nacalai tesque, Inc. (Kyoto, Japan). All other chemicals and reagents were of analytical grade. Melting points were determined on a Yanagimoto micro-melting point apparatus and are uncorrected. Optical rotations were measured with a JASCO DIP-1000 digital polarimeter. IR spectra were recorded on a Perkin-Elmer 1720X FTIR spectrophotometer. The ^1^H- (600 MHz) and ^13^C- (150 MHz) NMR spectra were recorded on an Agilent vnmrs600 instrument in CDCl_3_ with tetramethylsilane as the internal standard. The EIMS was recorded on a Hitachi 4000H double-focusing mass spectrometer (70 eV). Silica gel (70–230 mesh, Merck, Darmstadt, Germany) and silica gel 60 (230–400 mesh, Nacalai tesque, Inc.) were used for column chromatography and medium-pressure liquid chromatography, respectively. HPLC was carried out on an SiO_2_ column [Cosmosil 5SL-II column (Nacalai tesque, Inc.), 25 cm × 20 mm i.d.] at 25 °C with *n*-hexane/EtOAc [20:1 (HPLC system I), 10:1 (HPLC system II), and 5:1 (HPLC system III), flow rate 8.0 mL/min], and on ODS column [Cosmosil 5C_18_-MS-II column (Nacalai tesque, Inc.), 25 cm × 20 mm i.d.] at 25 °C with Me_2_CO:H_2_O [10:1 (HPLC system IV) and 9:1 (HPLC system V), flow rate 8.0 mL/min].

### 3.2. Plant Material

The seeds of *Cucurbita maxima*, produced in India, were purchased from Takada Seeds Co., Ltd. (Osaka, Japan) in 2011. A voucher specimen was deposited in the Herbarium of the Laboratory of Medicinal Chemistry, Osaka University of Pharmaceutical Sciences.

### 3.3. Extraction and Isolation

The seeds of *Cucurbita maxima* (10 kg) produced in India, were subjected to extraction with MeOH under reflux (30 L, one week, four times). The MeOH extract (310 g) was then partitioned between Et_2_O and H_2_O. The Et_2_O-soluble fraction (150 g) was subjected to SiO_2_ column chromatography (CC) [SiO_2_ (3.5 kg); CHCl_3_/MeOH 1:0, 10:1, 5:1, and 0:1 in increasing order of polarity] resulting in 9 fractions (Fr. A–I). Fr. B, eluted with CHCl_3_, was subjected to SiO_2_ CC to yield 18 fractions, B1–B18. Preparative HPLC of B7 (42.5 mg) (HPLC system II), eluted with hexane/EtOAc (10:1), gave **4** (28.0 mg; t_R_ 23.2 min). Fr. C, eluted with CHCl_3_, was subjected to SiO_2_ CC to yield 24 fractions, C1–C24. Preparative HPLC of C5 (67.9 mg)(HPLC system II), eluted with hexane/EtOAc (10:1), gave Fr. C5-4 (44.4 mg; tR 11.2 min), and then re-preparative HPLC gave **2** (11.1 mg; t_R_ 36.0 min)(HPLC system I). Preparative HPLC (HPLC system II) of C6 (64.0 mg), eluted with hexane/EtOAc (10:1), gave 15 fractions; C6-1–C6-15, and preparative HPLC (HPLC system II) of C7 (15.4 mg), eluted with hexane/EtOAc (10:1), gave 15 fractions; C7-1–C7-15. Preparative HPLC of C6-6 (2.9 mg; tR 13.0 min) and C7-6 (0.4 mg) combination gave **3** (1.0 mg; t_R_ 59.0 min)(HPLC system IV). Fr. E, eluted with CHCl_3_, was fractionated with SiO_2_ CC to E1–E11. Preparative HPLC (HPLC system III) of E8 (119.8 mg), eluted with hexane/EtOAc (5:1), gave Fr. E8-10 (8.8 mg; t_R_ 34.8 min), and then re-preparative HPLC (HPLC system V) gave 1 (1.6 mg; t_R_ 13.6 min).

### 3.4. Product Characterization Data

*7α-Hydroxymultiflor-8-ene-3α,29-diol 3-acetate-29-benzoate* (**1**): Colorless crystal (MeOH); mp 66–68 °C; 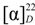 −85.8 (*c* = 0.2, CHCl_3_); UV (EtOH) λ_max_ (logε) 205.0 (3.80), 220.0 (3.94), 232.5 (3.98), 270.5 (3.41), 280.0 (3.32), 321.0 (2.87) nm; IR (KBr) ν_max_ 3437, 2938, 2876, 1718, 1272, 1244, 1110, 750, 729, 710 cm^−1^; ^1^H and ^13^C-NMR data see Table 1; EIMS *m/z* 586 [M–H_2_O]^+^ (26), 540 (33), 527 (40), 511 (100), 389 (12), 387 (9), 253 (15), 225 (16); HREIMS *m/z* 586.4019 (calcd for C_39_H_54_O_4_: 586.4023).

*7α-Methoxymultiflor-8-ene-3α,29-diol 3,29-dibenzoate* (**2**): Colorless crystal (MeOH); mp 88–90 °C; 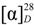 −20.7 (*c* = 0.7, CHCl_3_); UV (EtOH) λ_max_ (logε) 238.5 (3.70), 271.5 (3.21), 278.5 (3.11) nm; IR (KBr) ν_max_:2948, 2883, 1717, 1456, 1367, 1314, 1274, 1113, 1069, 716 cm^−1^;^1^H and ^13^C-NMR data see Table 1; EIMS *m/z* 680 (6) [M]^+^, 648 [M–MeOH]^+^ (12), 526 (25), 511 (100), 389 (8), 355 (10), 324 (8); HREIMS *m/z* 680.4447 (calcd for C_45_H_60_O_5_: 680.4441).

*7β-Methoxymultiflor-8-ene-3*α*,29-diol 3,29-dibenzoate* (**3**).Colorless crystal (MeOH); mp 67–68 °C; 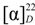 −43.7 (*c* = 0.2, CHCl_3_); UV (EtOH) λ_max_ (logε) 206.5 (3.86), 220.0 (3.97), 235.0 (4.00), 271.0 (3.29), 280.0 (3.16) nm; IR (KBr) ν_max_:3437, 1717, 1272, 1110, 1028, 975, 711 cm^−1^; ^1^H and ^13^C-NMR data see Table 1; EIMS 680 (65) [M]^+^, 665 (38), 648 [M–MeOH]^+^ (14), 526 (29), 511 (100), 393 (18), 381 (18), 354 (28); HREIMS *m/z* 680.4446 [M]^+^ (calcd for C_45_H_60_O_5_: 680.4440).

### 3.5. Cell Cultures

The cell lines HL-60 (human leukemia) and P388 (murine leukemia) were grown in RPMI 1640 medium, while B16 4A5 cells were grown in D-MEM. The medium was supplemented with 10% FBS and antibiotics (100 units/mL penicillin and 100 μg/mL streptomycin). The cells were incubated at 37 °C in a 5% CO_2_ humidified incubator.

### 3.6. Determination of B16 4A5 Cells Proliferation

The assay of B16 4A5 cells proliferation was examined according to a method reported previously [9]. 

### 3.7. Assay of Melanin Content

The assay of melanin content was performed as described previously [9].

### 3.8. Cytotoxicity Assay against Cancer Cell Lines

The cytotoxicity assay was determined previously [19].

## 4. Conclusions

In this study, we isolated three new multiflorane-type triterpene esters, *i.e.* 7α-hydroxymultiflor-8-ene-3α,29-diol 3-acetate-29-benzoate (**1**), 7α-methoxymultiflor-8-ene-3α,29-diol 3,29-dibenzoate (**2**), and 7β-methoxymultiflor-8-ene-3α,29-diol 3,29-dibenzoate (**3**), from pumpkin seeds. Isolated compounds were evaluated for melanogenesis inhibitory and cytotoxic activities. In the melanogenesis inhibitory assay, we revealed that compound **1** possessed melanogenesis inhibitory activities comparable with arbutin at a non-toxic concentration. In a cytotoxicity assay against cancer cell lines, none of the compounds showed remarkable cytotoxic activities. We will continue to explore other biological activities of multiflorane-type triterpenes.

## Supplementary Materials

Supplementary materials can be accessed at: http://www.mdpi.com/1420-3049/19/4/4802/s1.
