# Supplementary material for: Three New Triterpene Esters from Pumpkin (Cucurbita maxima) Seeds
Source: Molecules. 2014 Apr 16;19(4):4802–13. doi: 10.3390/molecules19044802 (PMC6271469; doi:10.3390/molecules19044802)
Supplement: Supplementary file 1 [file molecules-19-04802-s001.pdf]

Supporting Information

Figure S1. ^{1}H-NMR spectrum of compound **1**.

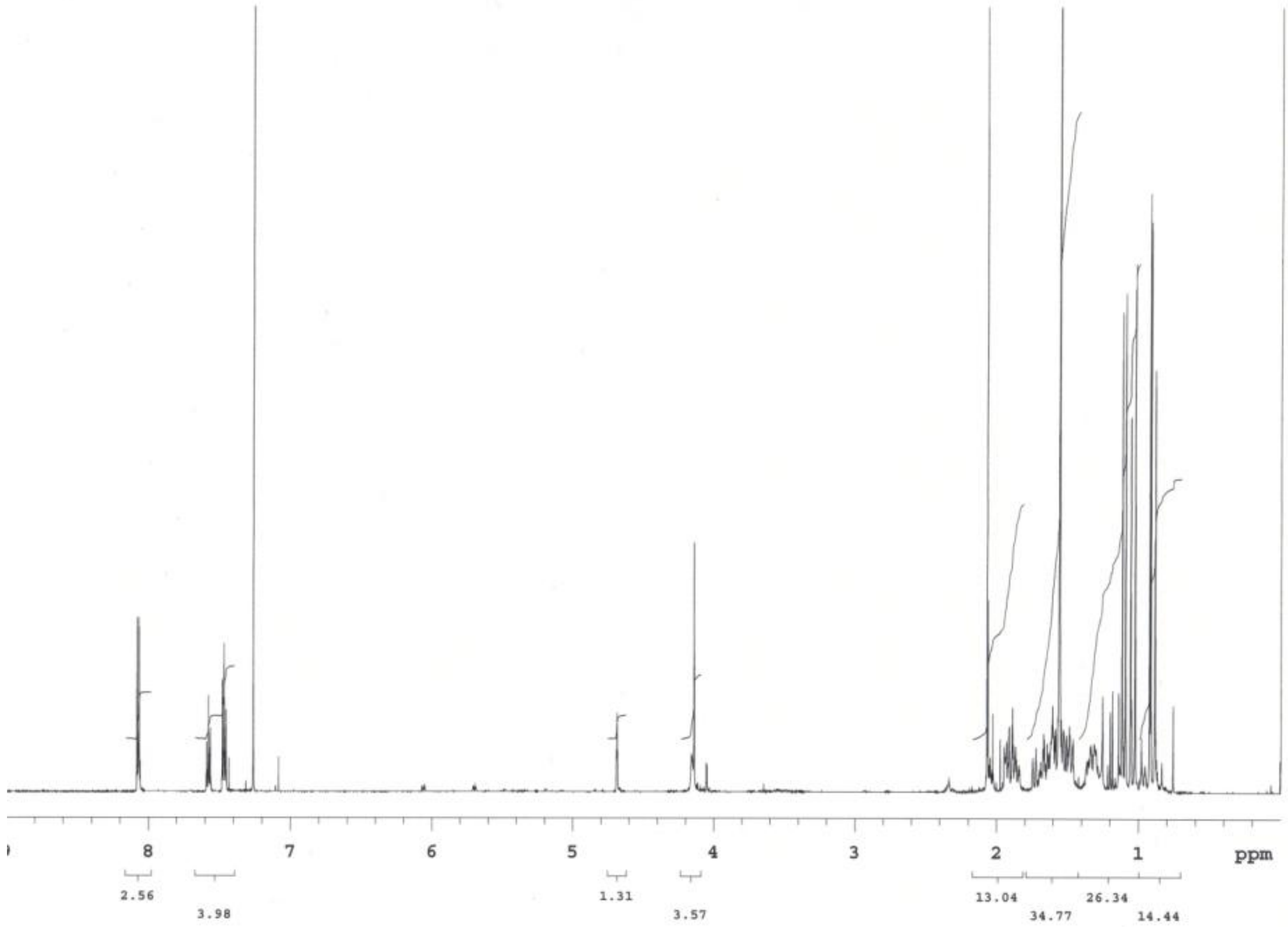

Figure S2. ^{13}C-NMR spectrum of compound **1**.

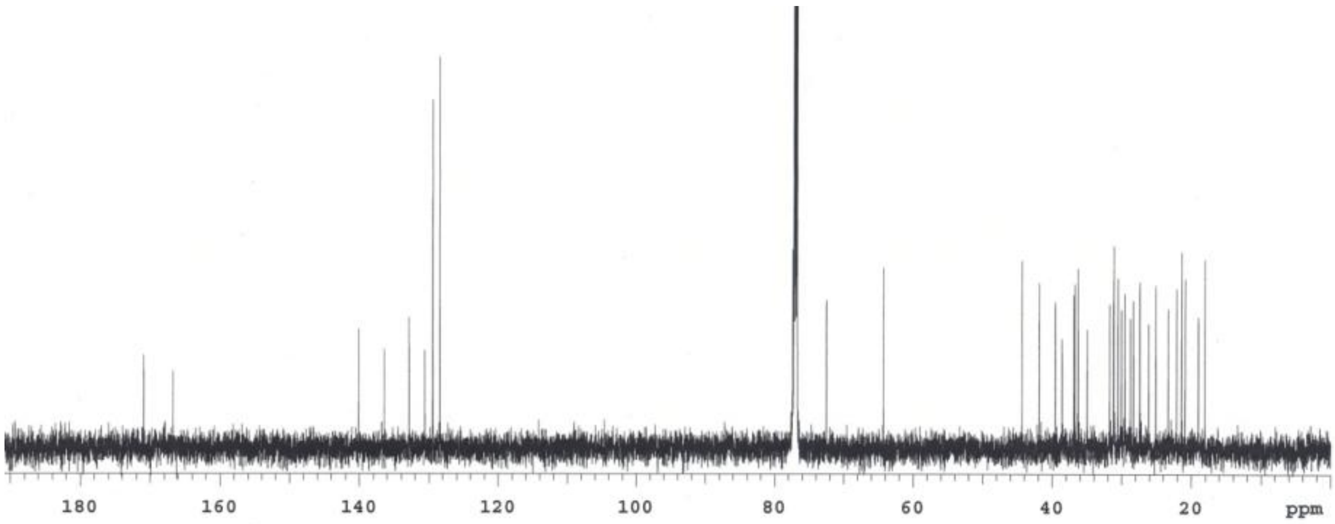

Figure S3. HSQC spectrum of compound **1**.

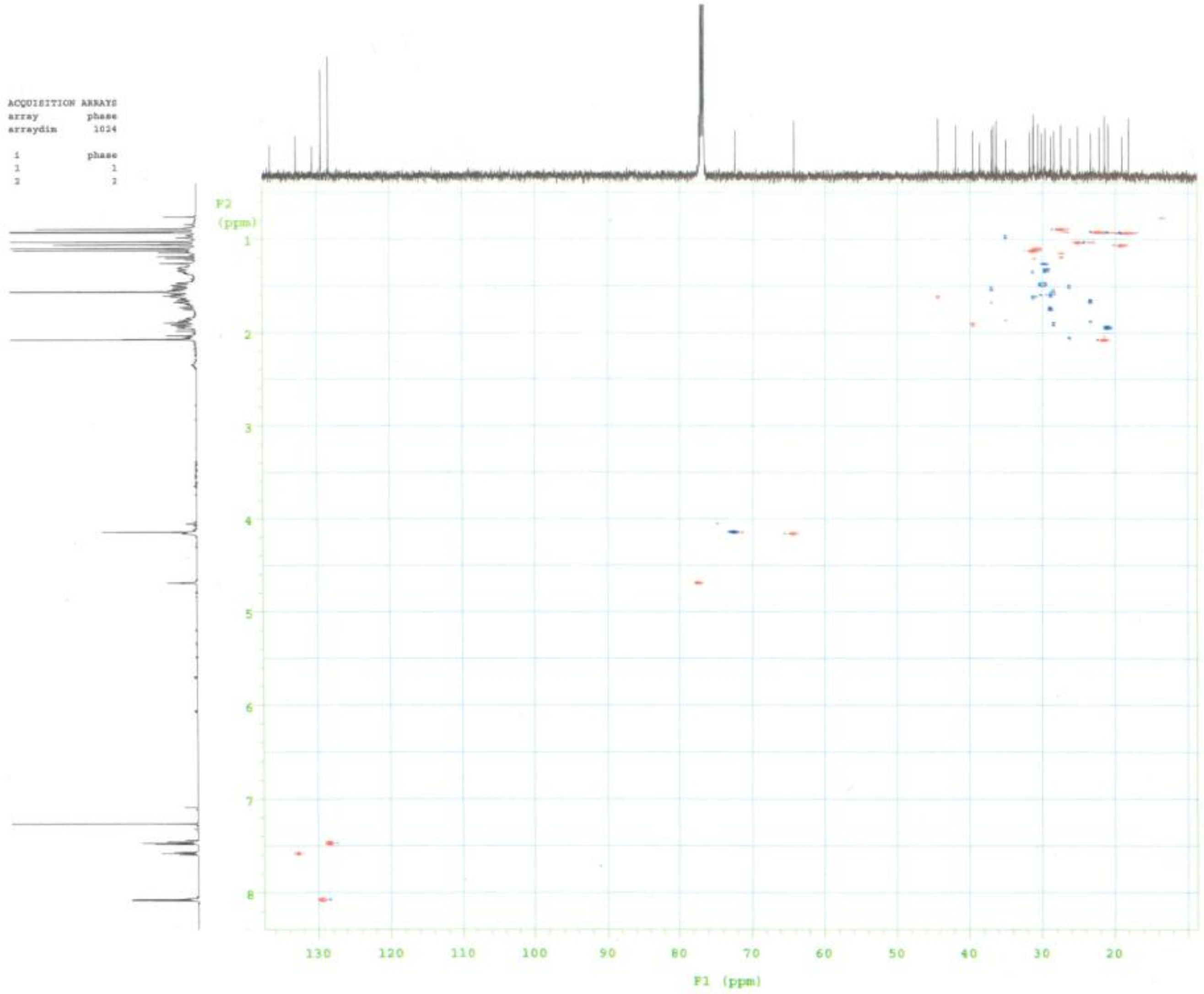

Figure S4. HMBC spectrum of compound **1**.

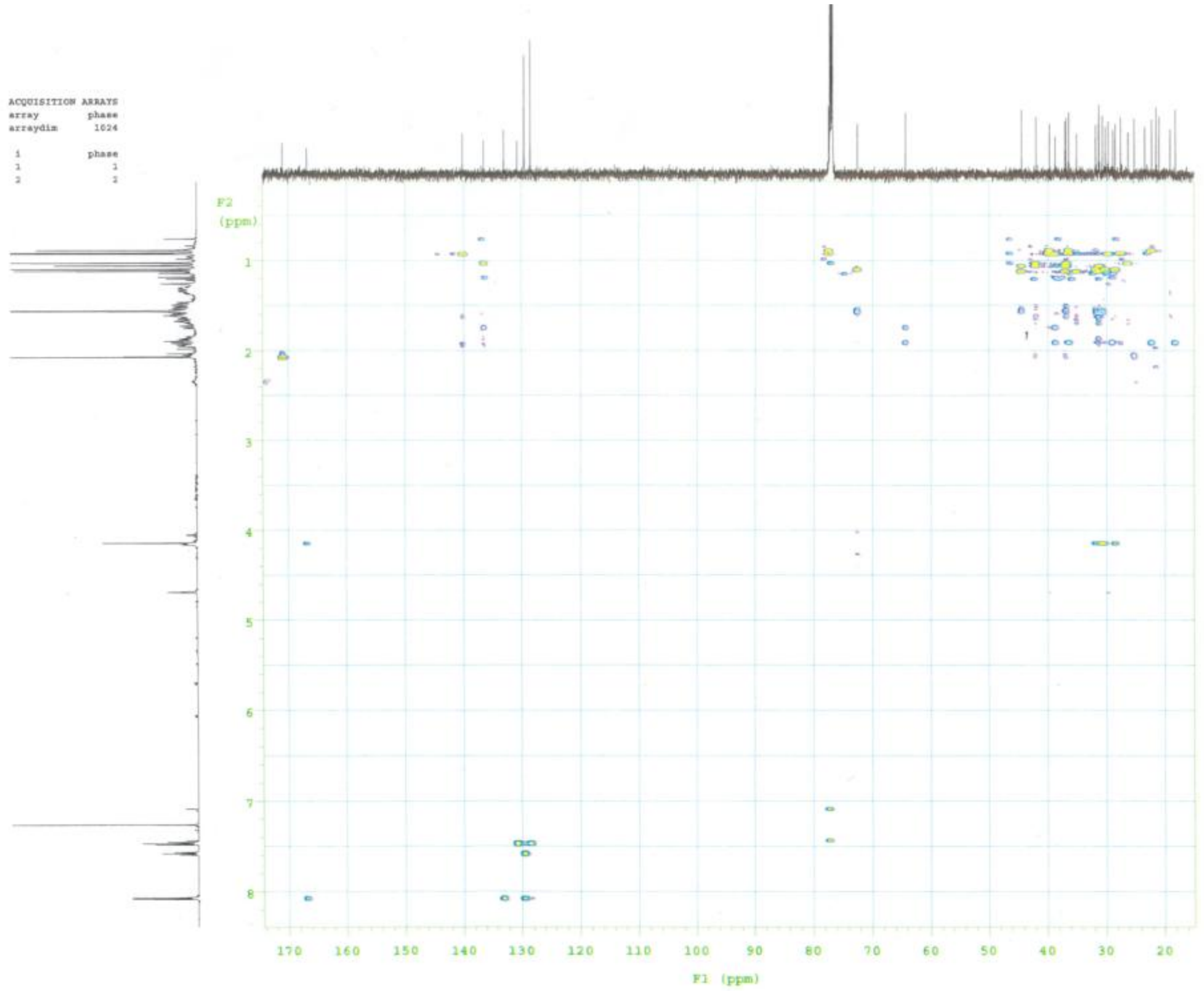

Figure S5. ^{1}H-^{1}H COSY spectrum of compound **1**.

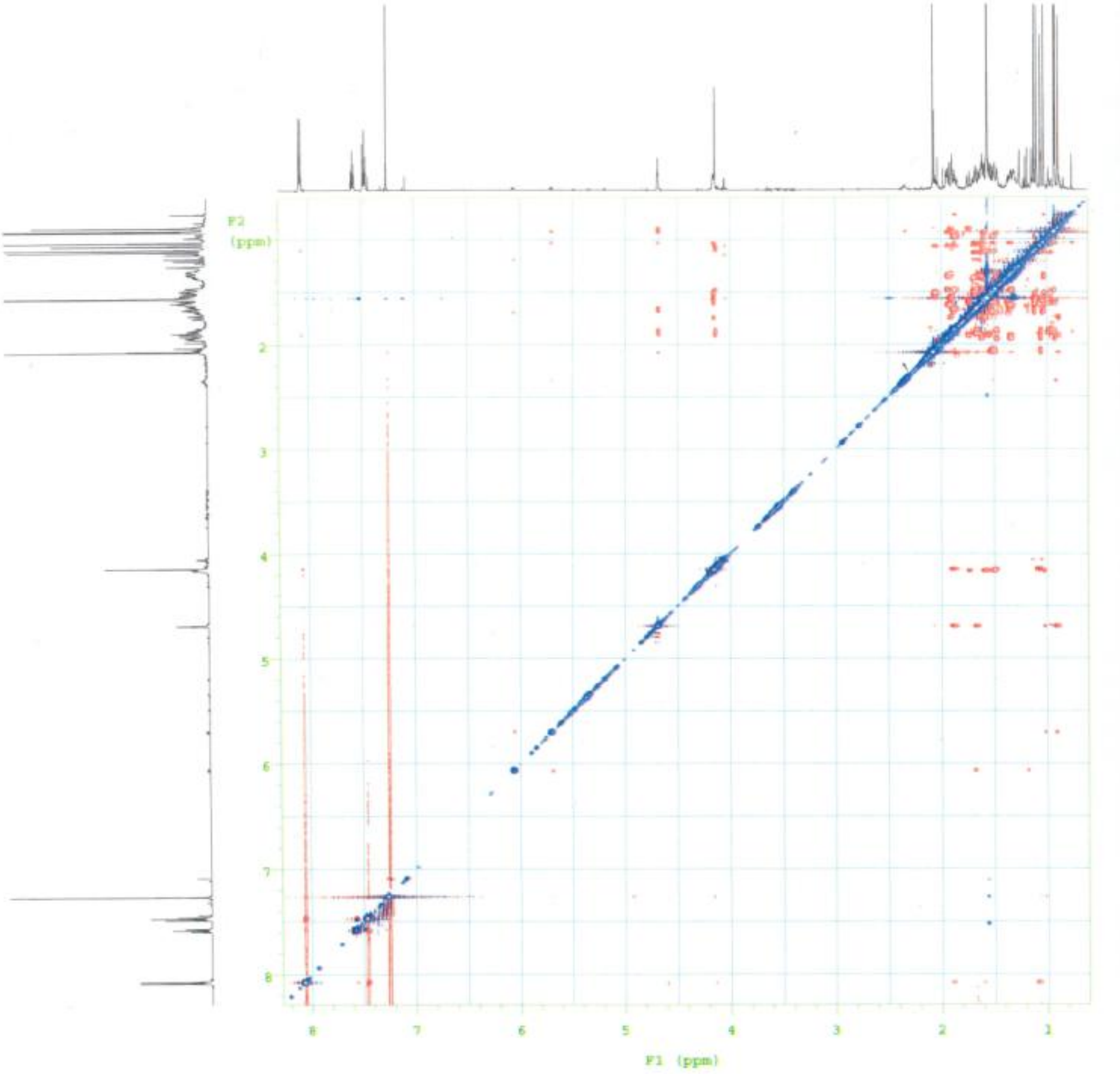

Figure S6. NOESY spectrum of compound **1**.

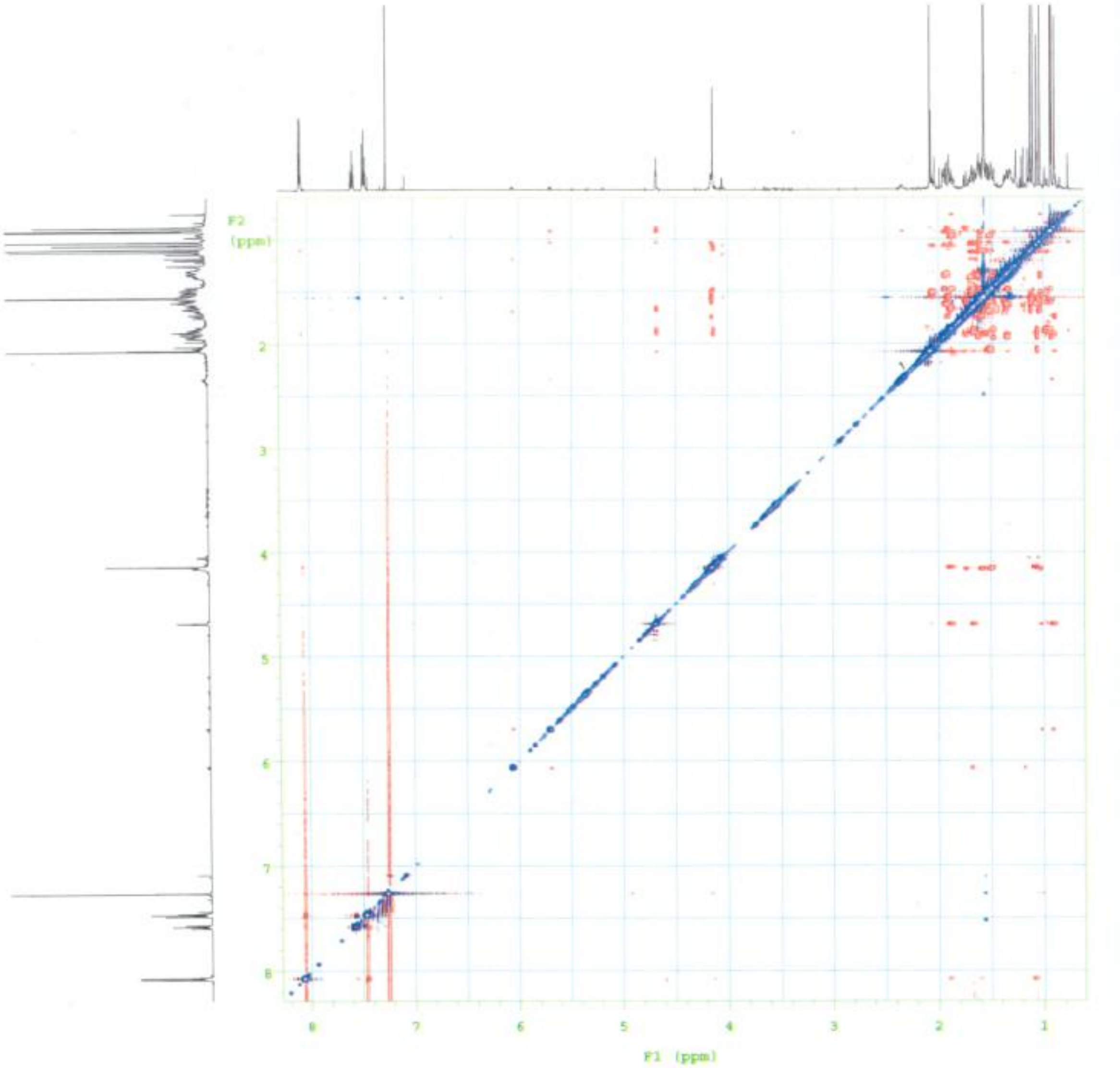

Figure S7. EI-MS of compound **1**.

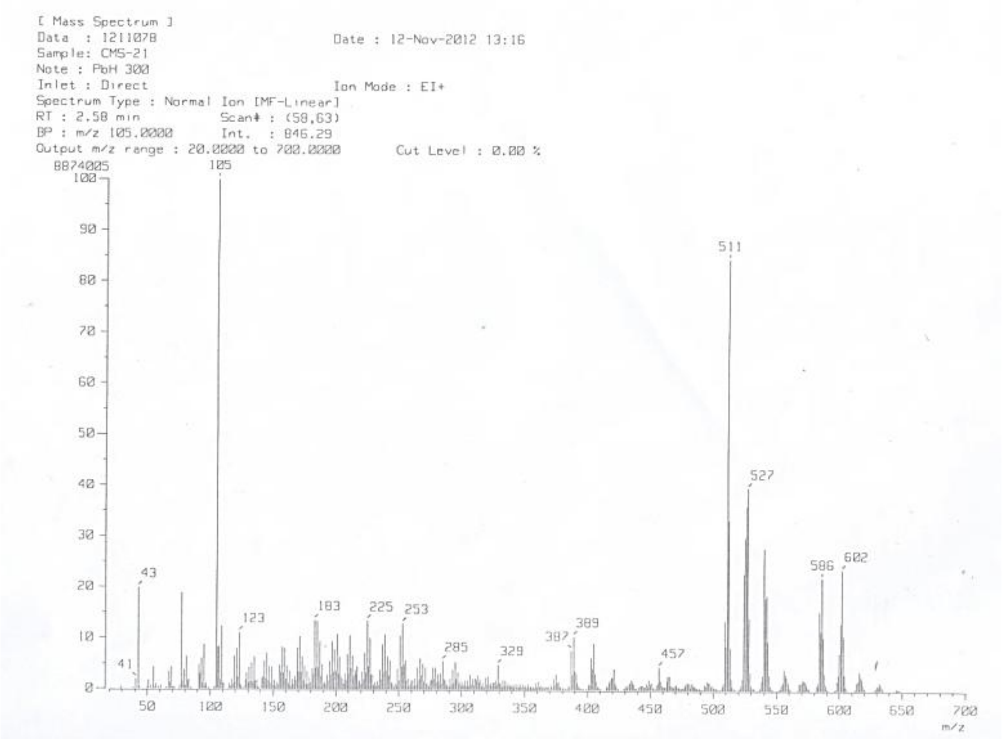

Table S1. ^{1}H (600 MHz) and ^{13}C (150 MHz), ^{1}H-^{1}H COSY, NOESY, and HMBC NMR Spectroscopic Data of Compounds **1**

position		δ$_H$ (*J* in Hz)	^{1}H-^{1}H COSY	NOE	δ$_C$, type	HMBC (H to C)
1	α	1.33, m	1β 2α 2β	5α	29.6, t	25
	β	1.47, m	1α 2α 2β			
2	α	1.66, m	1α 1β 2β 3β		23.3, t	
	β	1.88, m	1α 1β 2α 3β	24 25		
3		4.69, t (3.0)	2α 2β		77.4, d	23 24
4					36.3, s	5α 23 24
5		1.91, m	6α 6β	1α	39.6, d	23 24 25
6	α	1.74, m	5α 6β 7β	23	28.8, t	5α
	β	1.60, m	5α 6α 7β	24 25 26		
7		4.16, brs	6α 6β	15α 26	64.3, d	5α 6α
8					136.4, s	6α 11 26
9					140.1, s	11 12β 25
10					38.6, s	5α 6α 25
11		1.93, 2H, m	12α 12β		20.9, t	
12	α	1.34, m	11 12β		31.19, t	27
	β	1.61, m	11 12α	26		
13					36.8, s	12β 19β 26 27
14					41.9, s	12β 15α 15β 26 27
15	α	2.05, m	15β 16α 16β		26.1, t	16β 26
	β	1.50, m	16α 16β 15α	7β 27		
16	α	1.54, m	15α 15β 16β		36.9, t	15α 28
	β	1.67, m	15α 15β 16α	26		
17					31.2, s	16β 18 19α 19β 22α 28
18		1.61, m	19α 19β		44.4, d	16α 27 28
19	α	1.90, m	18β 19β		28.4, t	29 30
	β	1.56, m	18β 19α	28		
20					31.7, s	19β 29 30
21	α	1.47, m	21β 22α 22β		30.1, t	29 30
	β	1.59, m	21α 22α 22β	28		
22	α	1.87, m	22β 21α 21β	27 29	35.0, t	18 21β 28
	β	0.97, m	22α 21α 21β			
23		0.89, s		6α	27.4, q	5 24
24		0.91, s		2β 6β	22.1, q	5 23
25		0.92, s		2β 6β	18.1, q	5
26		1.03, s		7β 12β 16β 18	25.1, q	15α
27		1.06, s		15α 22α 29	19.0, q	12α 12β
28		1.12, s		19β 21β	31.17, q	
29		4.14, 2H, brs		27 22α	72.5, t	19β 30
30		1.10, s			30.6, q	19β 29
3-OCO					170.9, s	3 1'
1'		2.07, s			21.4, q	
29-OCO					166.7, s	29 2'',6''
1''					130.6, s	3'',5''
2'', 6''		8.07, dd (1.2, 7.4)	3'',5''		129.4, d	4'' 2'',6''
3'', 5''		7.47, tt (1.2, 7.4)	2'',6'' 4''		128.4, d	3'',5''
4''		7.58, tt (1.2, 7.4)	3'',5''		132.9, d	2'',6''

Figure S8. ^{1}H-NMR spectrum of compound **2**.

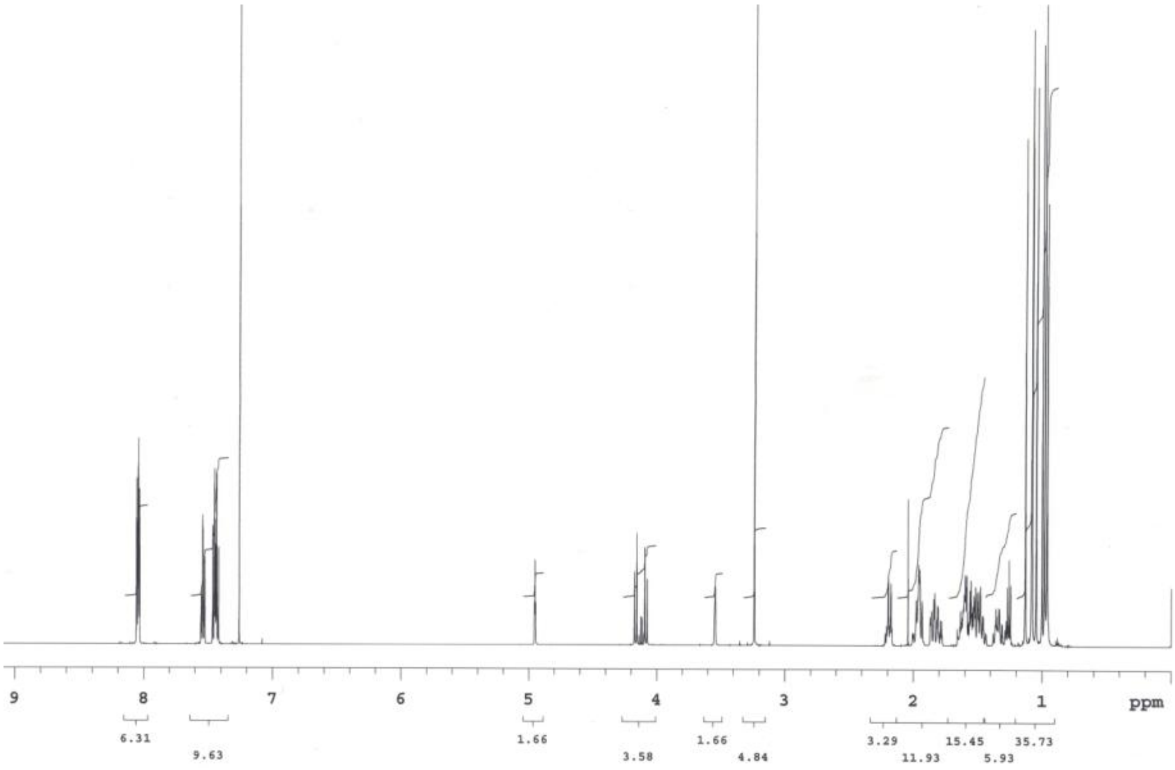

Figure S9. ^{13}C-NMR spectrum of compound **2**.

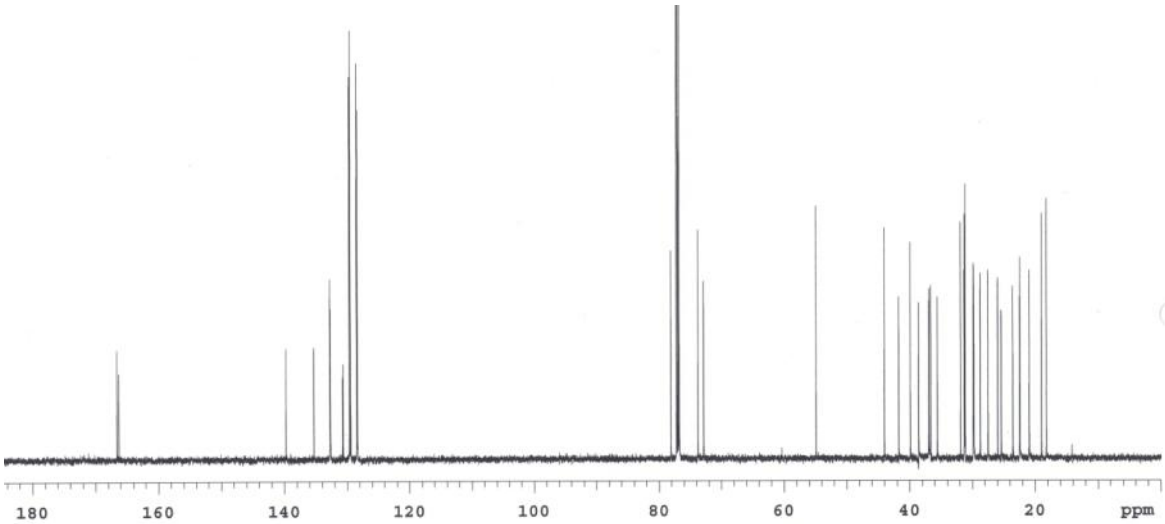

Figure S10. HSQC spectrum of compound **2**.

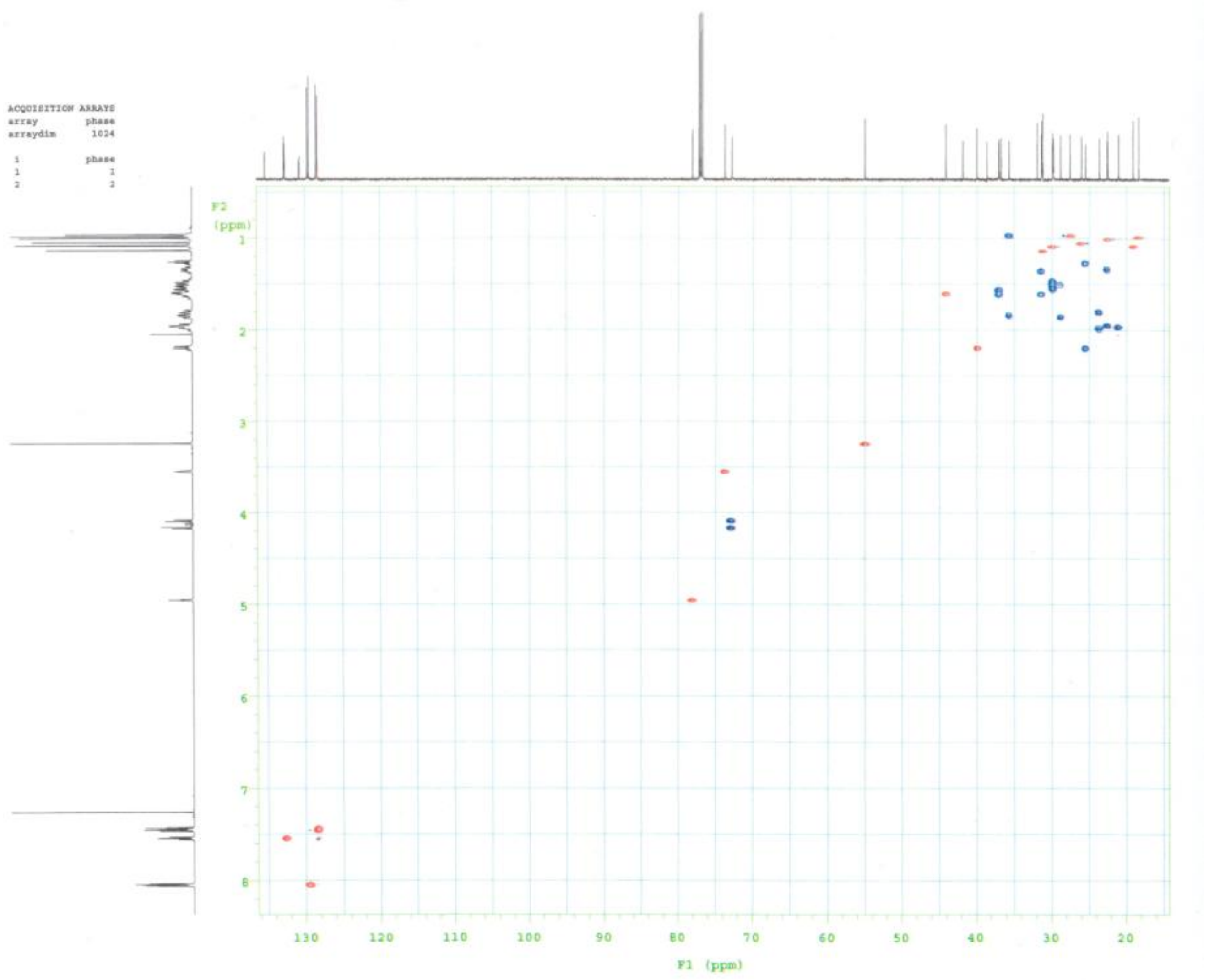

Figure S11. HMBC spectrum of compound **2**.

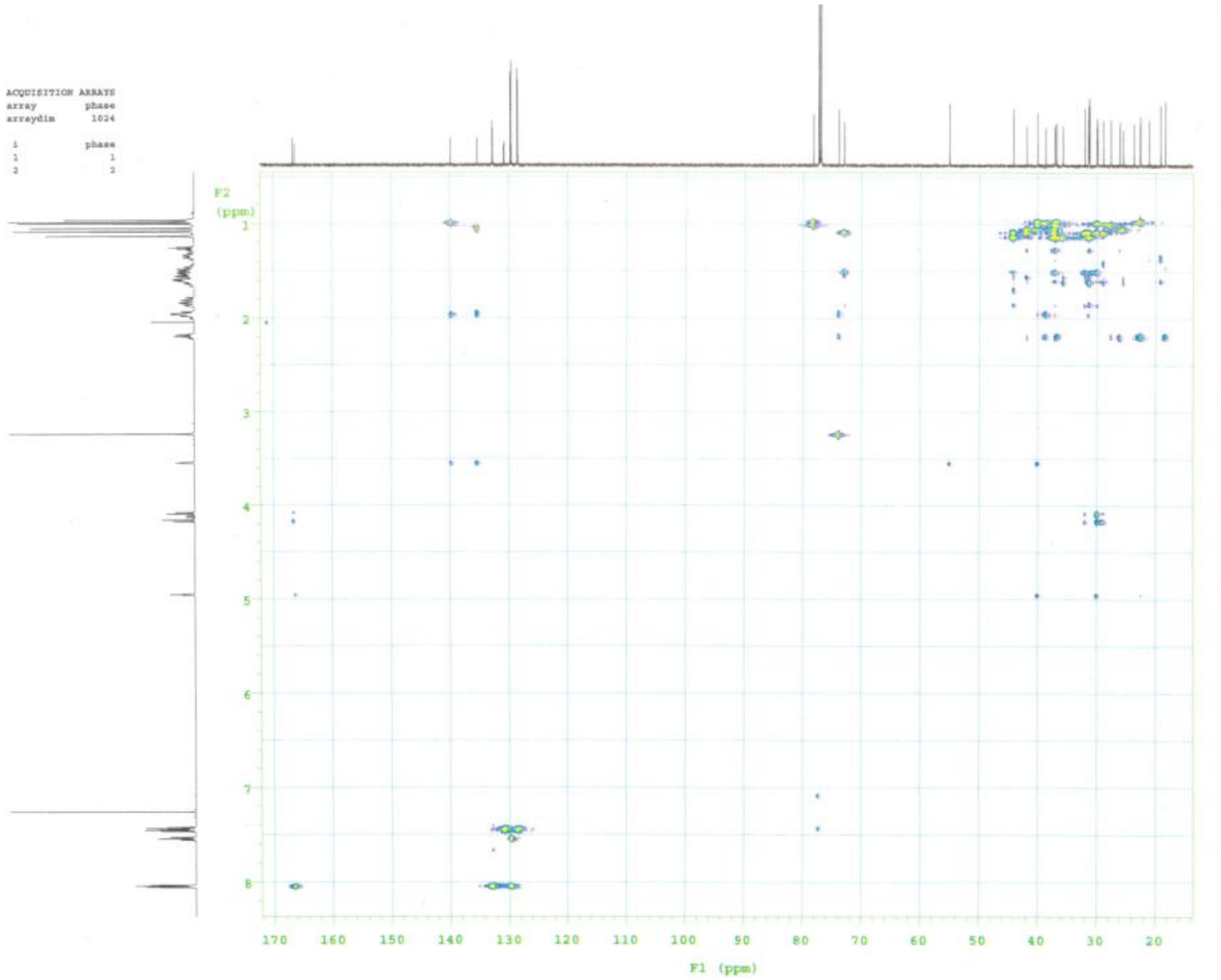

Figure S12. ¹H-¹H COSY spectrum of compound **2**.

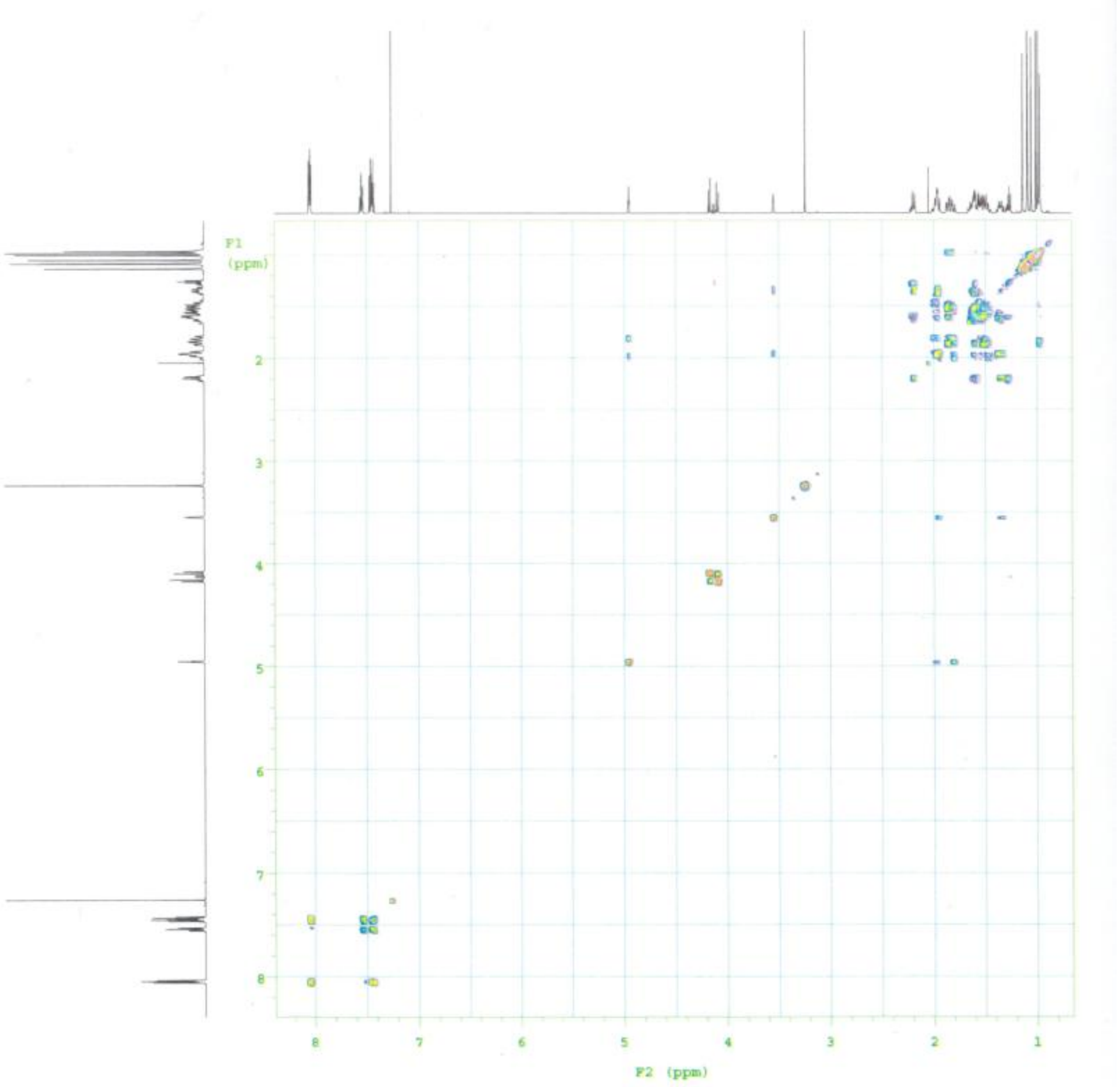

Figure S13. NOESY spectrum of compound **2**.

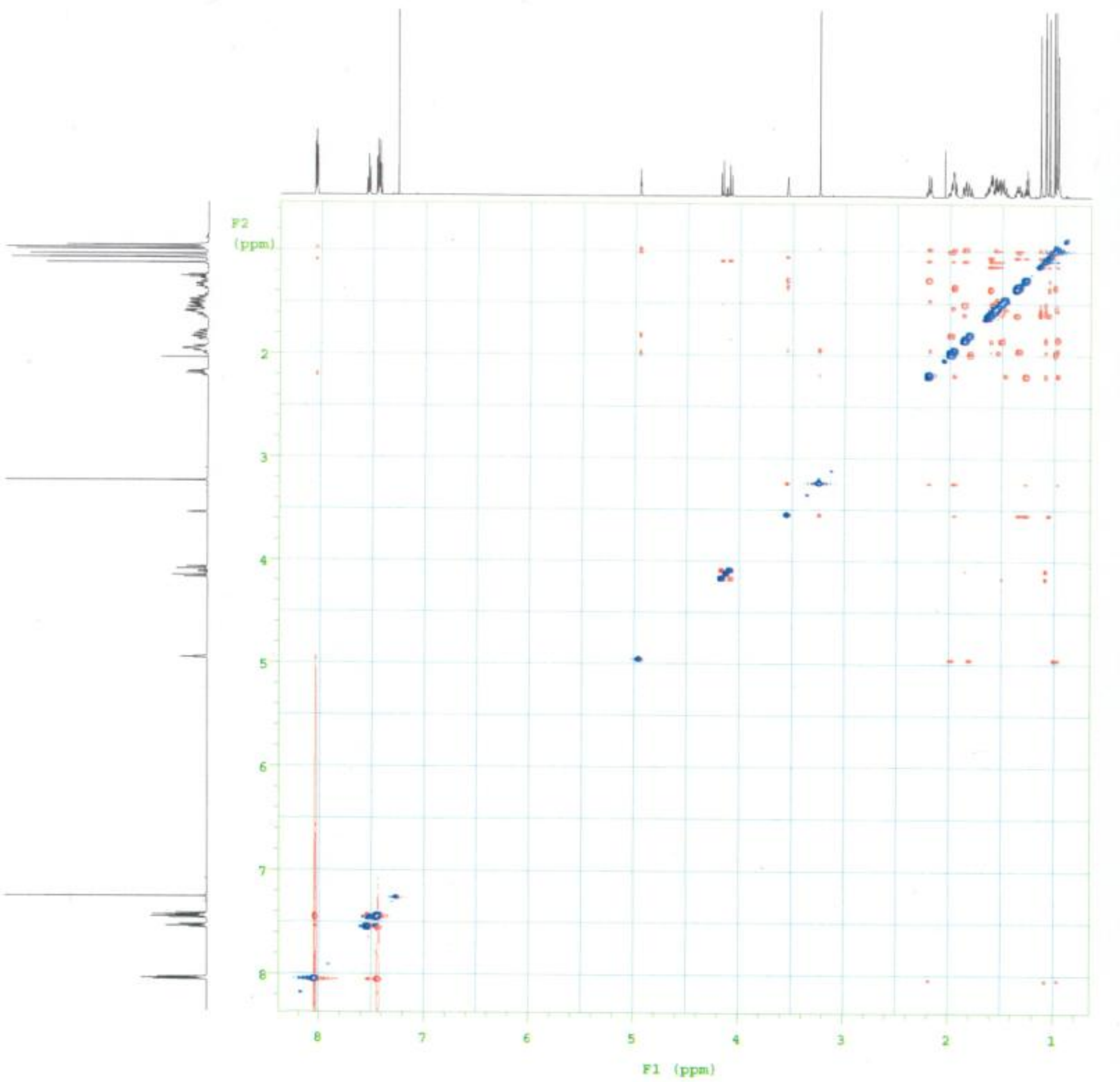

Figure S14. EI-MS of compound **2**.

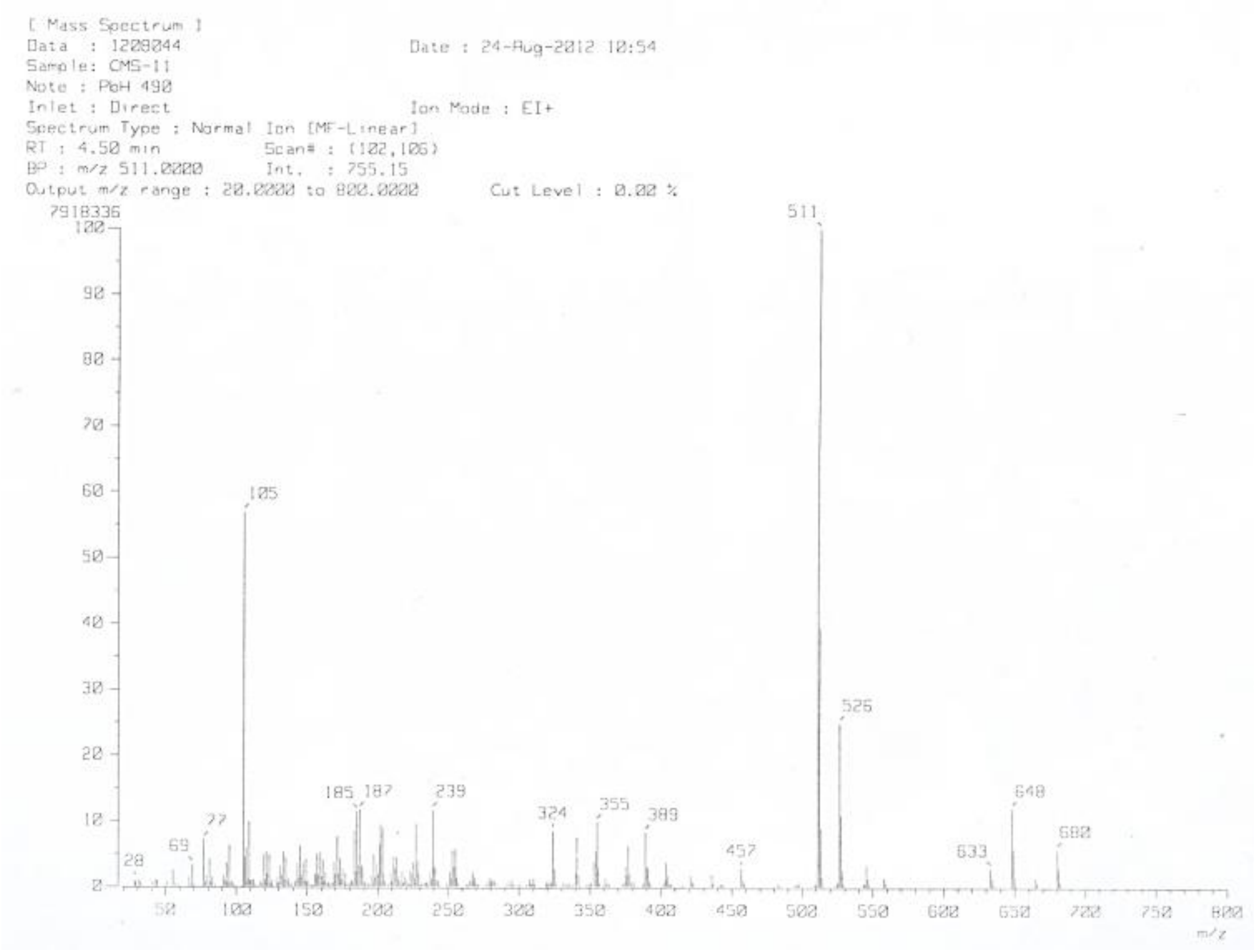

Table S2. ^{1}H (600 MHz) and ^{13}C (150 MHz), ^{1}H-^{1}H COSY, NOESY, and HMBC NMR Spectroscopic Data of Compounds **2**

position		δ_H (*J* in Hz)	^{1}H-^{1}H COSY	NOE	δ_C, type	HMBC (H to C)
1	α	1.48, m	1β 2α 2β	5α	29.8, t	3β 25
	β	1.53, m	1α 2α 2β			
2	α	1.80, m	1α 1β 3β		23.6, t	
	β	1.98, m	1α 1β 3β	24 25		
3		4.95, t (2.9)	2α 2β		78.1, d	23 24
4					36.7, s	5α 23 24
5		2.19, dd (1.2, 12.6)	6α 6β	1α 27 7-OMe	39.9, d	3β 6α 7β 23 24 25
6	α	1.95, m	5α 6β 7β	23	22.5, t	5α
	β	1.34, m	5α 6α 7β	24		
7		3.54, brs	6α 6β	15β 26	73.8, d	5α 6α
8					135.3, s	6α 7β 26
9					139.7, s	7β 11 25
10					38.6, s	5α 6α 25
11		1.97, m	12α 12β		20.9, t	12α
12	α	1.35, m	11 12β		31.3, t	11 27
	β	1.61, m	11 12α	26		
13					37.0, s	11 15β 18β 19α 19β 26 27
14					41.8, s	15α 15β 16β 26 27
15	α	2.19, m	15β 16α 16β	7-OMe	25.4, t	16α 26
	β	1.26, m	15α 16α 16β	7β		
16	α	1.56, m	15α 15β 16β		36.9, t	15β 22β 28
	β	1.61, m	15α 15β 16α	26		
17					31.1, s	15β 16α 16β 18 21α 22α 22β 28
18		1.60, m	19α 19β	26	44.0, d	16β 21β 22α 27 28
19	α	1.86, m	18β 19β		28.8, t	18β 29a 29b 30
	β	1.49, m	18β 19α	28		
20					31.9, s	19β 22α 29a 29b 30
21	α	1.48, m	21β 22α 22β		29.9, t	19β 22α 22β 29a 29b 30
	β	1.53, m	21α 22α 22β	28		
22	α	1.84, d (4.4)	21α 21β 22β	27	35.6, t	16α 16β 18 28
	β	0.96, m	21α 21β 22α			
23		0.97, s		6α	27.5, q	5 24
24		1.00, s		2β 6β 25	22.4, q	5 23
25		0.98, s		2β 24	18.2, q	5
26		1.05, s		7β 12β 16β 18	26.0, q	15α 15β
27		1.082, s		5α 22α 29a 29b	19.0, q	12α 12β 18
28		1.13, s		19β 21β	31.3, q	16α 16β 18 22α 22β
29	a	4.16, d (10.8)		27	72.9, t	19β 21α 21β 30
	b	4.08, d (10.8)		27		
30		1.084, s			29.8, q	29a 29b
3-OCO					166.3, s	3 2',6'
1'					130.8[a], s	3',5'
2", 6"		8.05[a], dd (1.4, 7.4)	3',5'		129.6[b], d	4' 2',6'
3", 5"		7.45[b], tt (1.4, 7.4)	2',6' 4'		128.4[c], d	4'
4"		7.55[c], tt (1.4, 7.4)	3',5'		132.7[d], d	3',5'
29-OCO					166.6, s	29a 29b 2", 6"
1"					130.7[a], s	3",5"
2", 6"		8.04[a], dd (1.4, 7.4)	3",5"		129.4[b], d	2",6" 4"
3", 5"		7.43[b], tt (1.4, 7.4)	2",6" 4"		128.3[c], d	4"
4"		7.54[c], tt (1.4, 7.4)	3",5"		132.6[d], d	2",6"
7-OMe		3.24, s		5α 15α	54.9, q	

a—d Interchengeable.

Figure S15. ^{1}H-NMR spectrum of compound **3**.

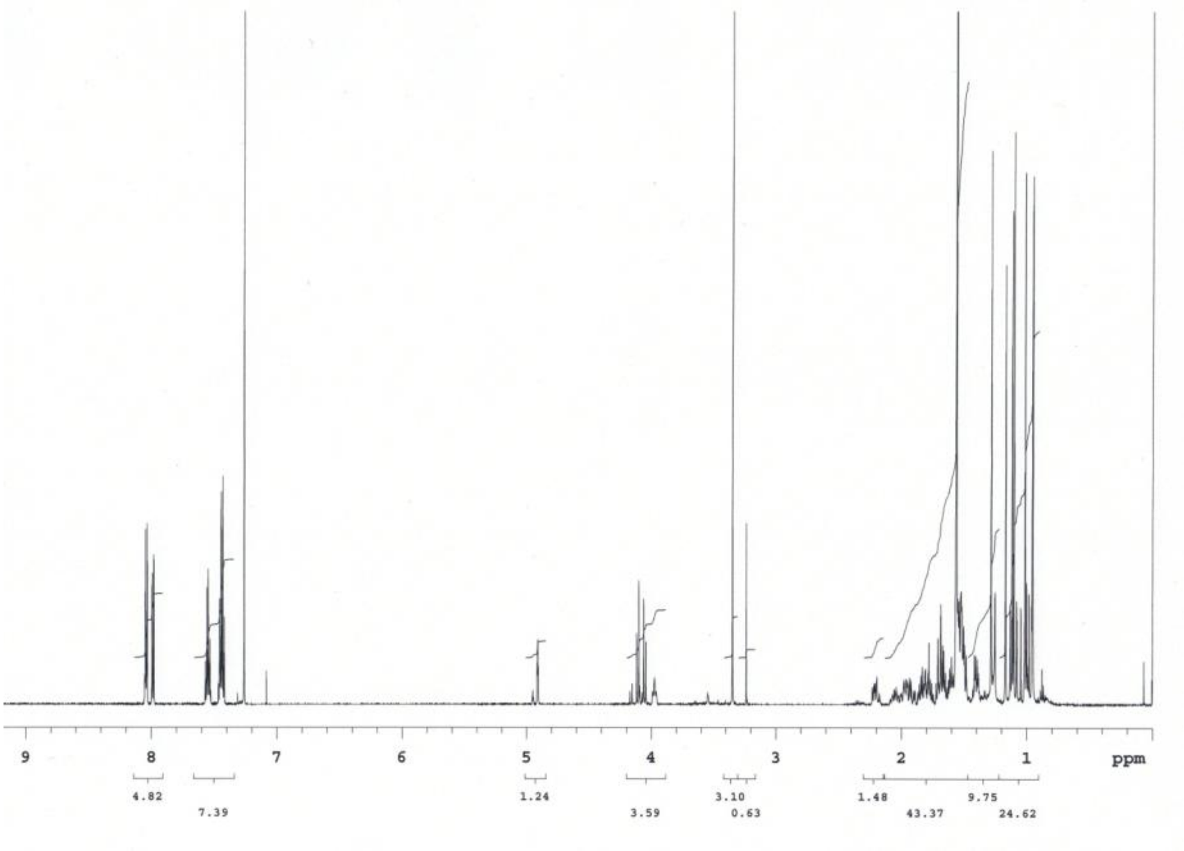

Figure S16. ^{13}C-NMR spectrum of compound **3**.

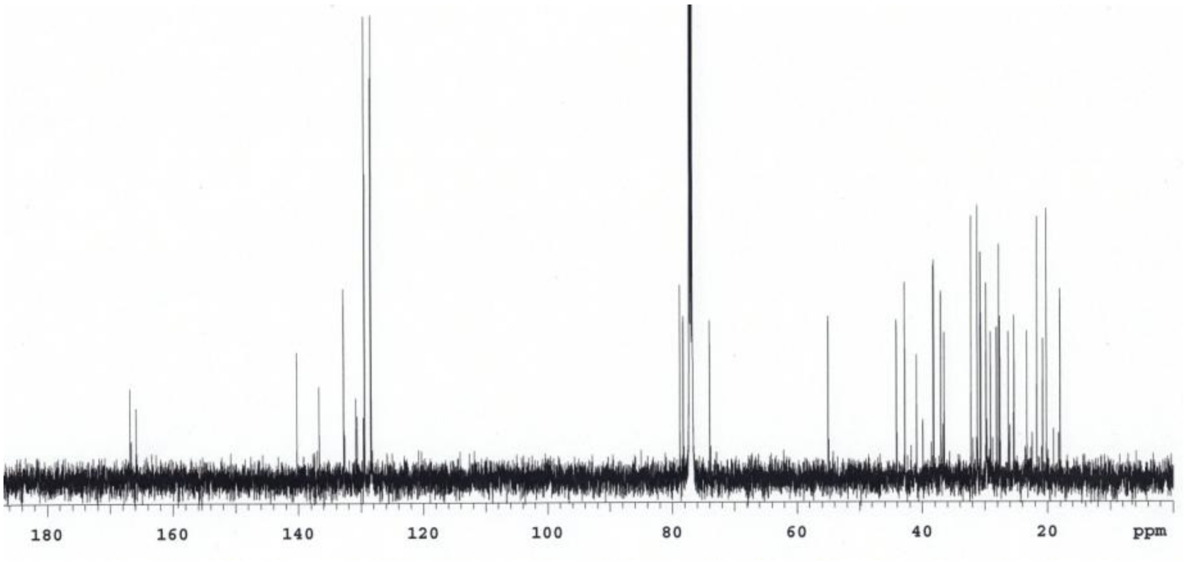

Figure S17. HSQC spectrum of compound **3**.

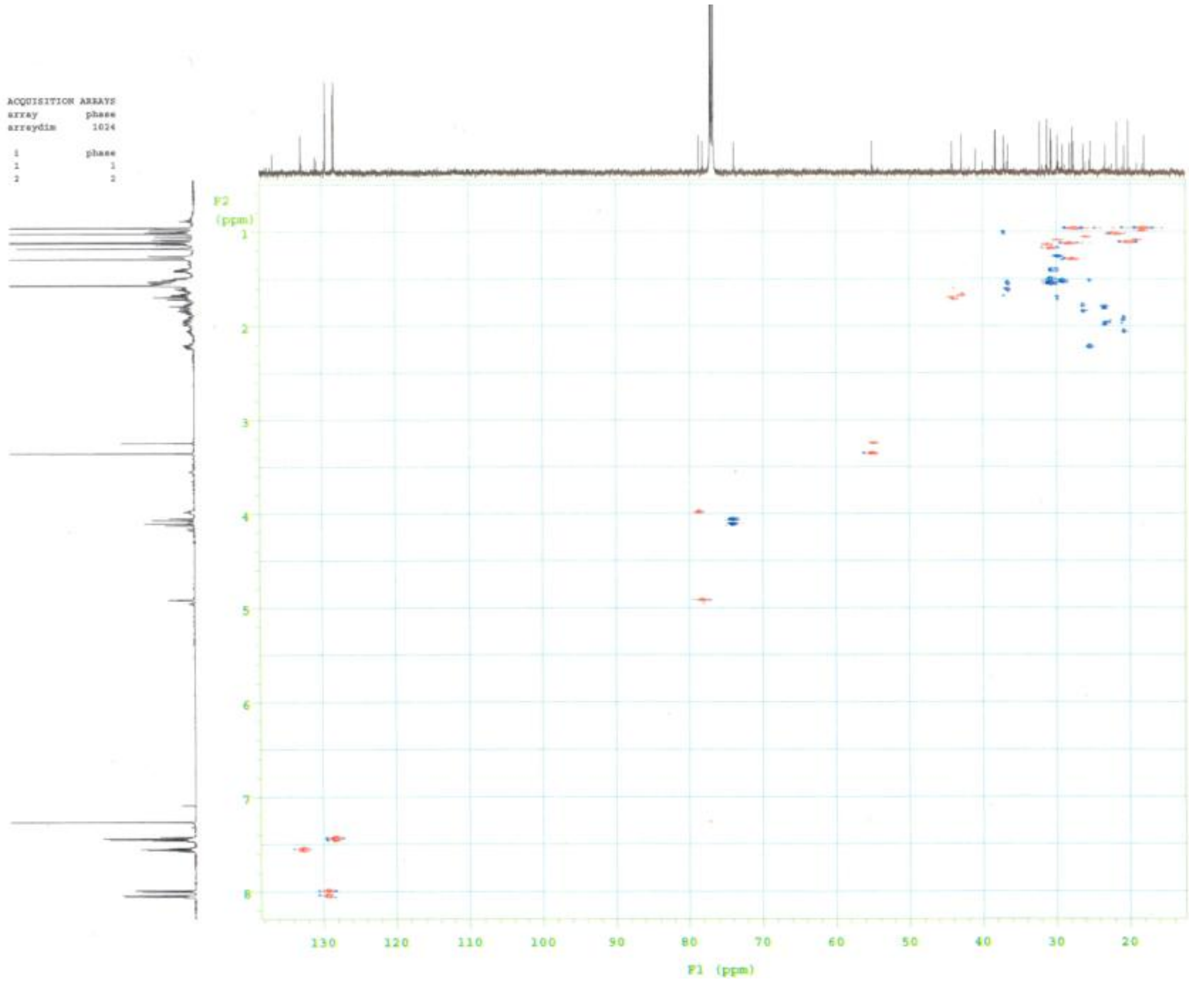

Figure S18. HMBC spectrum of compound **3**.

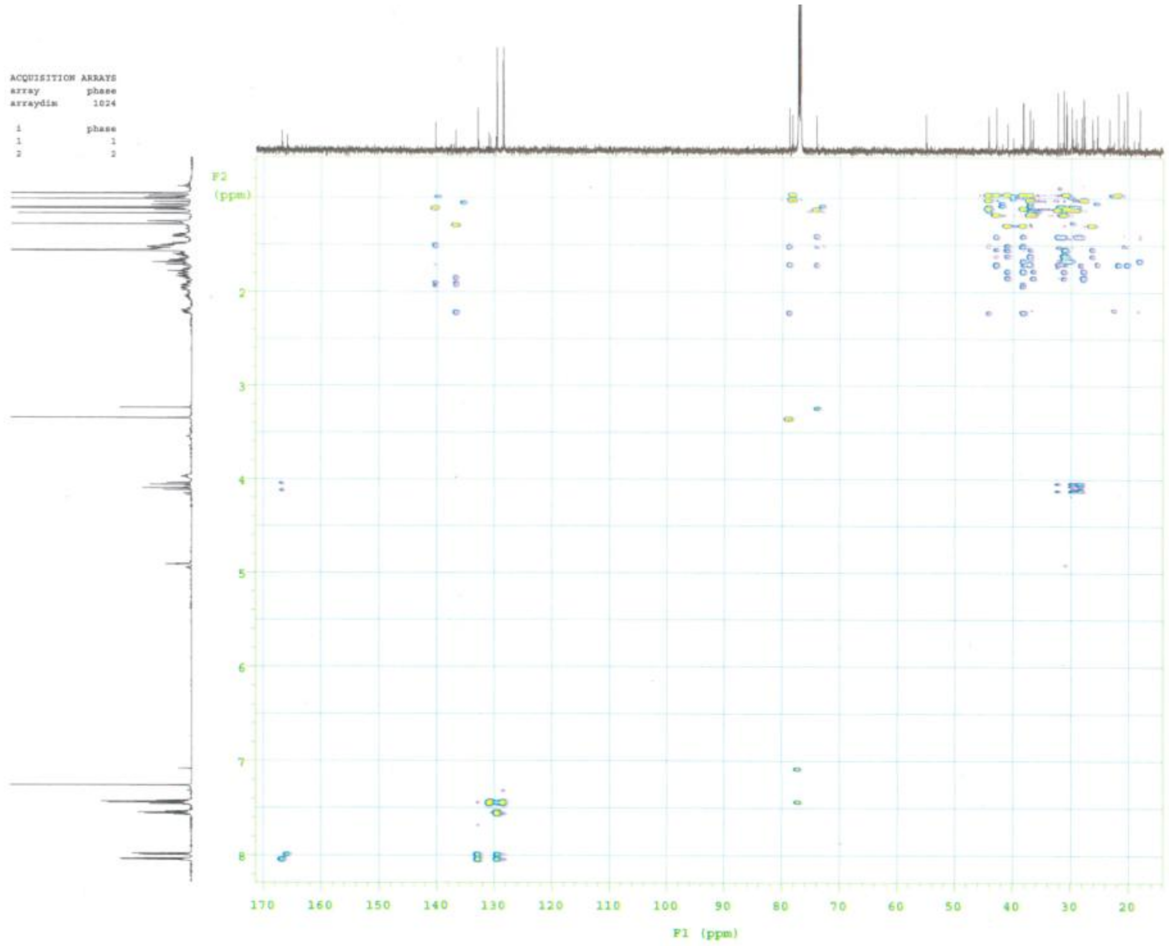

Figure S19. ^{1}H-^{1}H COSY spectrum of compound **3**.

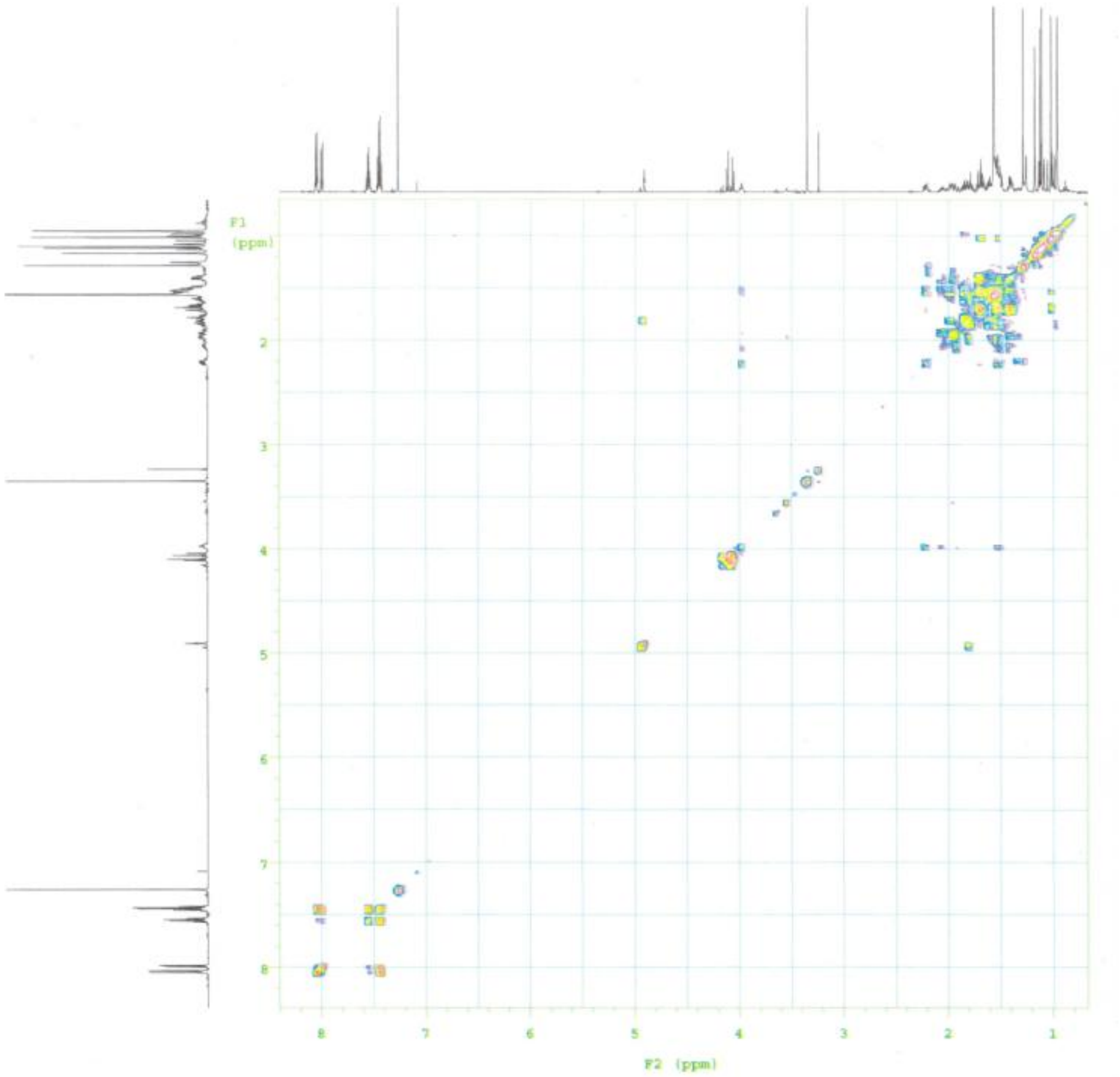

Figure S20. NOESY spectrum of compound **3**.

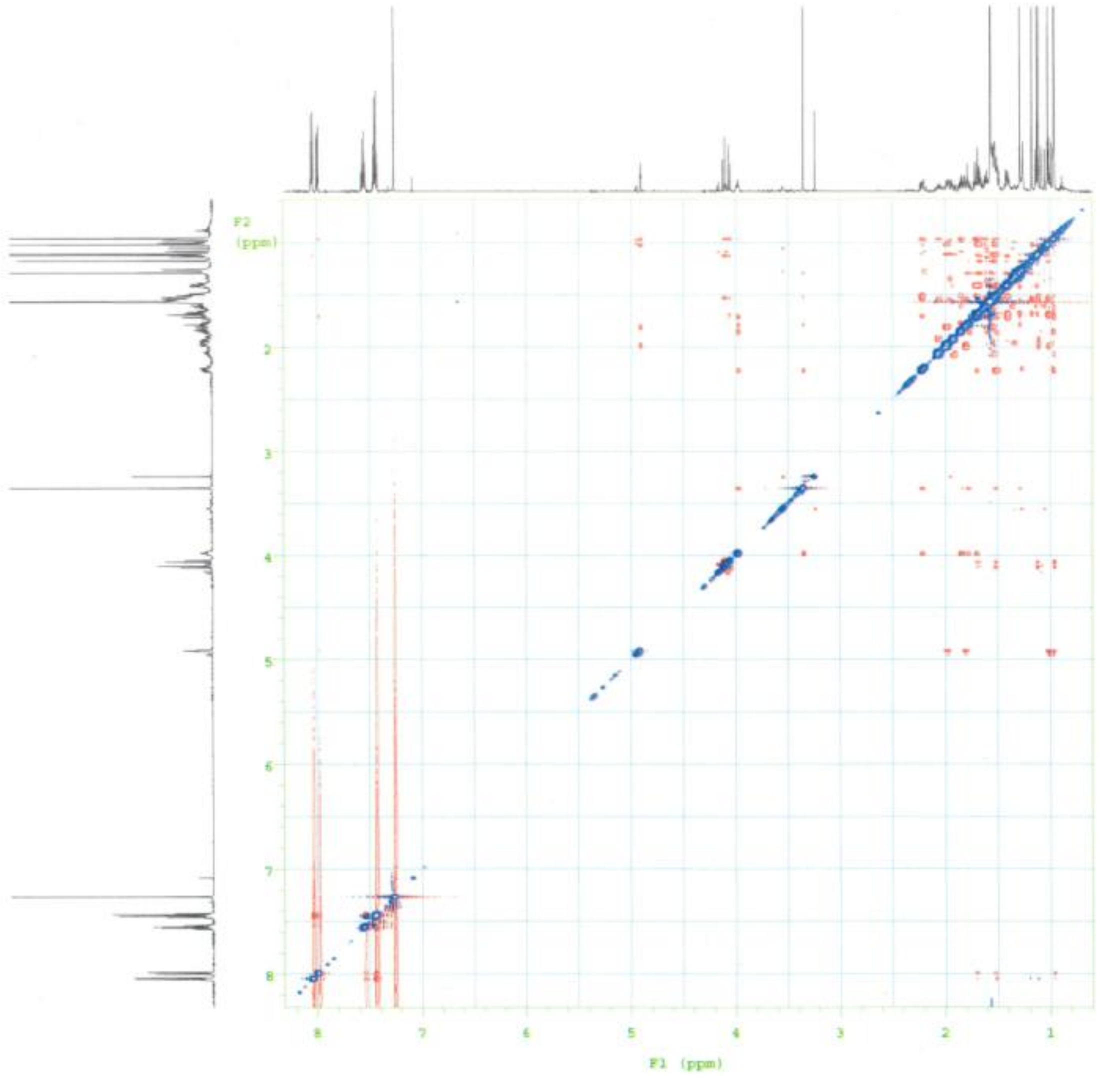

Figure S21. EI-MS of compound **3**.

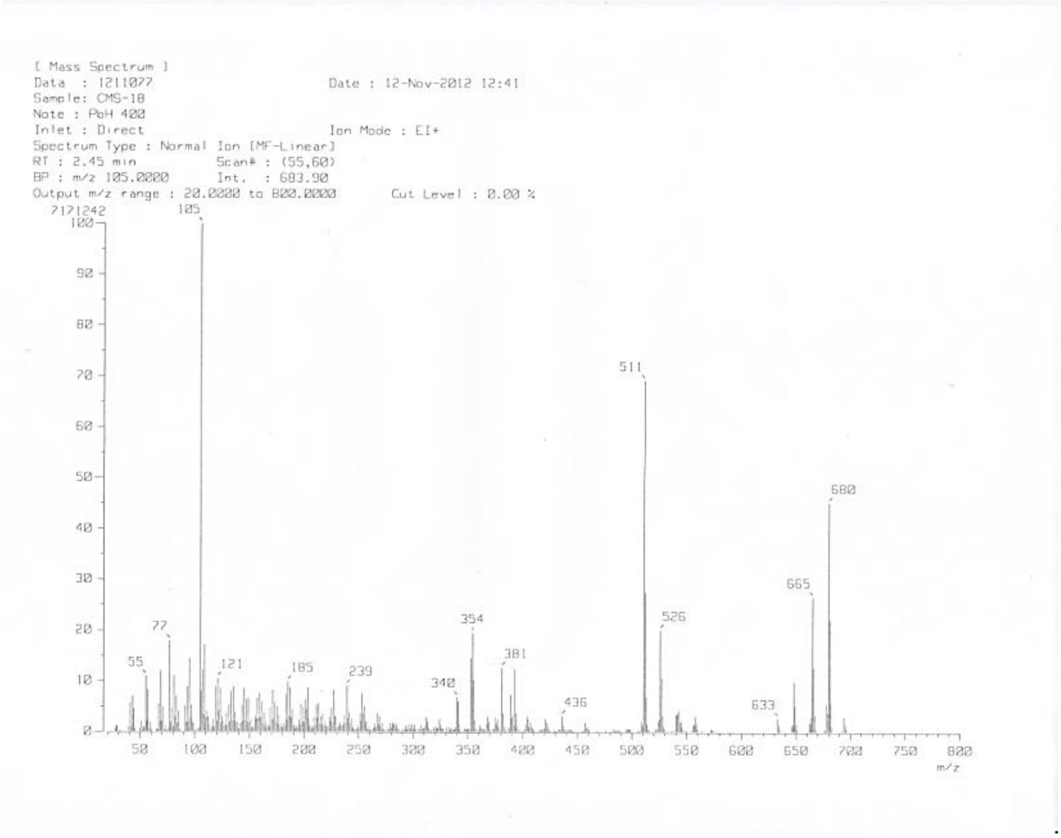

Table S3. ^{1}H (600 MHz) and ^{13}C (150 MHz), ^{1}H-^{1}H COSY, NOESY, and HMBC NMR Spectroscopic Data of Compounds **3**

position		δ_H (*J* in Hz)	^{1}H-^{1}H COSY				NOE				δ_C, type	HMBC (H to C)					
1	α	1.40, m	1β	2α	2β		5α				30.6, t	25					
	β	1.52, m	1α	2α	2β												
2	α	1.80, m	1α	1β	2β	3β					23.3, t						
	β	1.97, m	1α	1β	2α	3β	24	25									
3		4.91, t (3.0)	2α	2β							78.2, d	23	24				
4											37.0, s	5α	23	24			
5		1.70, m	6α	6β			1α	7α			44.1, d	6α	23	24	25		
6	α	2.21, m	5α	6β	7α						25.3, t	5α					
	β	1.51, m	5α	6α	7α												
7		3.98, brt (7.6)	6α	6β			5α	15α			78.8, d	5α	6α	6β	7-OMe		
8											136.7, s	7α	11α	11β	15β	26	
9											140.3, s	1β	11β	25			
10											38.2, s	1α	1β	5	6α	25	
11	α	2.03, m	11β	12α	12β		27				20.8, t	12α					
	β	1.91, m	11α	12α	12β												
12	α	1.51, m	11α	11β	12α						30.7, t	18β	27				
	β	1.40, m	11α	11β	12β		26										
13											38.2, s	26					
14											40.9, s	15α	15β	16α	16β	26	27
15	α	1.78, m	15β	16α	16β		7α				26.3, t	16α	16β	26			
	β	1.83, m	15α	16α	16β												
16	α	1.61, m	15α	15β	16β						36.5, t	15α	15β	28			
	β	1.53, m	15α	15β	16α		18β										
17											31.2, s	15α	15β	19α	28		
18		1.66, m	19α	19β			16β	26			42.8, d	16β	19α	27	28		
19	α	1.40, m	18β	19α	19β						29.8, t	18β	29a	29b	30		
	β	1.50, m	18β	19α	19β		28										
20											32.2, s	19α	19β	22β	29a	29b	30
21		1.52, m	22α	22β							29.1, t	29a	29b	30			
22	α	1.68, m	21	22β			27				37.1, t	16α	16β	21	28		
	β	1.01, m	21	22α													
23		0.96, s					6α				27.6, q	24					
24		1.02, s					6β				21.7, q	5α	23				
25		1.10, s					2β				20.2, q	5α					
26		1.29, s					12β	18β	7-OMe		27.8, q	15α	15β				
27		0.95, s					11α	22α	29a	29b	18.0, q	12β	18β				
28		1.17, s					19β	26			30.7, q						
29	a	4.11, d (10.6)					27				74.0, t	19α	30				
	b	4.05, d (10.6)					27										
30		1.12, s									28.1, q	19α	29a	29b			
3-OCO											165.9, s	3β	2',6'				
1'											130.6, s						
2'', 6''		7.99, dd (1.4, 7.4)	3',5'								129.4, d	2',6'	4'				
3'', 5''		7.45, tt (1.4, 7.4)	2',6'	4'							128.5, d	3',5'					
4''		7.56, tt (1.4, 7.4)	3',5'								132.8, d	2',6'					
29-OCO											166.8, s	29a	29b	2'',6''			
1''											130.9, s						
2'', 6''		8.04, dd (1.7, 7.3)	3'',5''								129.5, d	2'',6''	4''				
3'', 5''		7.43, tt (1.7, 7.3)	2'',6''	4''							128.4, d	3'',5''					
4''		7.55, tt (1.7, 7.3)	3'',5''								132.8, d	2'',6''					
7-OMe		3.35, s					26				55.0, q						
